# Bacterial microbiome analysis of vaginal, cervical, and endometrial samples in patients with adenomyosis during the window of implantation

**DOI:** 10.1128/spectrum.02791-25

**Published:** 2026-02-18

**Authors:** Nika Troha, Tomaž M. Zorec, Lea Hošnjak, Katja Strašek Smrdel, Andraž Celar Šturm, Vesna Šalamun, Snježana Grazio Frković, Eda Vrtačnik Bokal, Mario Poljak

**Affiliations:** 1 Faculty of Medicine, University of Ljubljana 37664, Ljubljana, Slovenia; 2 Department of Human Reproduction, Division of Obstetrics and Gynecology, University Medical Center Ljubljana 37667, Ljubljana, Slovenia; 3 Institute of Microbiology and Immunology, Faculty of Medicine, University of Ljubljana 37664, Ljubljana, Slovenia; Cleveland Clinic Lerner Research Institute, Cleveland, Ohio, USA

**Keywords:** adenomyosis, reproductive tract, microbiome, *Lactobacillus iners*, *Lactobacillus gasseri*, *Gardnerella vaginalis*, window of implantation, infertility, reproductive health

## Abstract

Adenomyosis is a chronic gynecological condition affecting a substantial portion of women of reproductive age. With symptoms including abnormal uterine bleeding, chronic pelvic pain (CPP), dysmenorrhea, and infertility, poor response to symptomatic treatment and unfavorable outcomes of assisted reproductive technologies, it remains a significant diagnostic and therapeutic challenge. Here, we analyzed microbial compositions of the reproductive tract of women with adenomyosis (*n* = 33) in comparison with healthy controls (*n* = 31). Vaginal, cervical, and endometrial samples were collected using minimally invasive transcervical sampling techniques on the 22nd day of the menstrual cycle, during the so-called window of implantation. 16S rRNA was amplified and recorded using the next generation sequencing. Bioinformatic and statistical analysis focused on quantitative taxonomical characterization of the specimens’ microbiomes. Vaginal bacterial microbiome composition was consistent across the three anatomical sites. Compared with the control group, adenomyosis was associated with *Lactobacillus iners,* whereas *Lactobacillus gasseri* and *Gardnerella vaginalis* were negatively associated with adenomyosis and its clinical symptoms. *G. vaginalis*, typically considered a pathogen, was highlighted as an important dominant microbiome replacement for lactobacilli, more so in healthy women than in women with adenomyosis. *Anaerococcus prevotii*, *Peptoniphilus grossensis,* and *Peptrostreptococcus anaerobius* also showed weak correlation to adenomyosis. Differences in taxa abundance were detected in association with adenomyosis clinical symptoms. *L. iners* was associated with dysmenorrhea, heavy menstrual bleeding (HMB), as well as CPP. *Prevotella disiens, Prevotella timonesis,* and *Dialister micraerophilus* were associated with dysmenorrhea and *Peptoniphilus grossensis* with HMB, respectively. *L. gasseri* and *L. jensenii* appeared to anticorrelate with these symptoms.

**IMPORTANCE:**

Adenomyosis poorly responds to treatment and assisted reproductive technologies. Here, we report a comprehensive 16S rRNA-based analysis of vaginal, cervical, and endometrial samples, obtained minimally invasively (transcervically) in a cohort of Caucasian women during the receptive phase of endometrium. Results revealed the least invasive option, vaginal microbiome sampling, reliably predicts the microbiome compositions of cervix and endometrium. We showed substantial variation in microbial composition of adenomyosis patients. *L. iners*, a species with specific functional traits, was consistently associated with adenomyosis presence and related symptoms. This finding suggests microbiome remodeling as a viable novel therapeutic option for adenomyosis. Furthermore, our findings indicate that the pathogenic role of *G. vaginalis* may be context-dependent. Ongoing genomic and ecological profiling is essential to clarify *Gardnerella's* dual commensal-pathogenic nature. Previous adenomyosis studies have mostly focused on the vaginal microbiome, whereas the endometrial microbiome has rarely been studied and never in the time of window of implantation.

## INTRODUCTION

Adenomyosis is a chronic gynecological condition characterized by the presence of endometrial glands and/or stroma in myometrium (1). With the advancements of imaging diagnostics, there is increasing evidence that adenomyosis occurs in 20%–35% of women of reproductive age (2). Adenomyosis presents with abnormal uterine bleeding, dysmenorrhea, chronic pelvic pain (CPP), infertility, and perinatal complications (3). Etiopathogenesis of adenomyosis remains unknown. It is likely a multifactorial condition involving multiple intricate pathophysiological mechanisms. These include disruption of the endometrial-myometrial interface with increased endometrial invasion into the myometrium, metaplasia of stem or germ cells within the myometrium, and implantation of ectopic tissue driven by alterations in local and systemic steroid and pituitary hormone regulation. Additionally, aberrant immune response and genetic and epigenetic modifications may play critical roles (4–6).

Using next-generation sequencing technology, recent research has shown that anatomical regions of the female reproductive tract (RT) previously considered sterile (e.g., the uterine cavity and the upper part of the RT) may also be inhabited by diverse microorganisms (7, 8). The dominance of lactobacilli and the resulting low microbial diversity in the vaginal microbiome have consistently been associated with favorable reproductive and perinatal outcomes. On the other hand, vaginal dysbiosis, e.g., bacterial vaginosis (BV) has been associated with preterm birth, late spontaneous and recurrent miscarriages (9–11), as well as susceptibility to sexually transmitted infections, pelvic inflammatory disease, endometritis, possibly resulting in infertility (12). A higher abundance of vaginal lactobacilli seems to be associated with lower concentrations of pro-inflammatory immune markers in the vaginal fluid (11, 13). On the contrary, no consensus exists on the composition of healthy endometrial microbiota or even the existence of a core microbiome (14, 15). Studies have indicated poor clinical outcomes in assisted reproductive technology (ART) procedures in the presence of endometrial dysbiosis (16–18).

For endometriosis, a condition similar to adenomyosis characterized by the presence of ectopic endometrial tissue, the so-called “bacterial contamination hypothesis” has been proposed. Altered microbiota may exacerbate pathological uterine contractions, facilitating the retrograde dissemination of endometrial tissue into the peritoneal cavity (19) and potentially cause DNA damage, mutations, and epithelial cell dysfunction (20). Considering the complex and enigmatic pathophysiology of adenomyosis with underlying inflammatory and hyperestrogenic metabolic conditions, a disturbance in RT microbiota has been proposed in adenomyosis etiopathogenesis (21–23). The meaning of the bacterial colonizers of RT and their effect on endometrial receptivity or exacerbation of disease symptoms remains unclear but could be associated with aberrant local metabolic conditions or modified host immune response. Microorganisms may promote the release of proinflammatory mediators, compromise the immune surveillance, and alter the immune cell profiles (abnormal activation and differentiation of dendritic cells, macrophages, and natural killer cells) (24, 25) and change the tissue remodeling pathways (26), all of which might impact embryo implantation and worsen symptoms of adenomyosis (27, 28).

Previous research that focused mainly on vaginal microbiome showed that patients with adenomyosis exhibit vaginal dysbiosis more often than controls (22, 24, 27). Research on microbial composition beyond the vagina in adenomyosis patients is scarce. A pilot study researching the uterine cavity microbiome in samples post-hysterectomy (study conducted in a population with median age 45 years) showed distinct microbial profiles, altered metabolic pathways, and suggested *C. freundii*, *P. copri*, and *B. cepacia* as potential pathogenic microorganisms associated with adenomyosis. Valdez Bango et al. showed distinct bacterial compositions in gut and vaginal microbiota in adenomyosis patients and enrichment of endometrial microbiota with family *Ruminococcaceae* and genus *Actinomyces* in the adenomyosis group.

Here, we assessed and compared vaginal, cervical, and endometrial microbiomes of women with adenomyosis to age-matched healthy controls. We aimed to identify bacterial taxa that could serve as specific adenomyosis biomarkers or would appear in association with adenomyosis-related clinical signs and could be screened as non-invasively as possible. Since the female RT is considered a temporally dynamic econiche (29), we were particularly interested in a possible role of microorganisms during the receptive phase of the menstrual cycle, the so-called window of implantation, during which relevant interaction might occur between the host metabolism of endometrium, the reproductive microbiota, and the embryo.

## RESULTS

### Demographic characteristics of the recruited cohort

A total of 33 women with adenomyosis and 31 healthy controls were included in this study. The two study groups showed no notable difference in participant age, body mass index, parity, number of deliveries, miscarriages, or ART success rates (Table 1). However, women with adenomyosis were more frequently diagnosed with infertility. Sonographic and clinical characteristics of the adenomyosis group are additionally shown in Table 1.

**TABLE 1:** Demographic data of control and study group^*a*^

Characteristic	Control group, *N* = 31	Adenomyosis group, *N* = 33	*P*-value
Age	35 (31, 36.5)	36 (33, 39)	0.099
BMI	23 (20.5, 27.2)	23.2 (20.65, 27.65)	0.827
Nulliparity	14 (45.1%)	19 (57.6%)	0.45
Number of deliveries	0 (0, 2)	0 (0, 1.75)	0.54
Number of spontaneous miscarriages	0 (0, 1)	0 (0, 1)	0.593
Chronic endometritis	0	2 (6.2%)	0.5
ART treatment	4 (13%)	17 (51.5%)	0.005
Successful pregnancy after ART	3 (75%)	11 (64.7%)	1.0
Diffuse adenomyosis	–	28 (84.8%)	–
Focal adenomyosis	–	5 (15%)	–
Enlarged or globular uterus	–	13 (82.2%)	–
Chronic pelvic pain	0	7 (21.9%)	–
Heavy menstrual bleeding	3 (10.3%)	19 (57.6%)	–
Dysmenorrhea	0	15 (46.9%)	–

^*a*^ BMI, body mass index. Data are presented in medians and with 25th and 75th percentiles or *n* (%). “–” indicates that the clinical finding or symptom was not observed in the control group and therefore was not evaluated.

### Samples were sequenced at adequate depth

Swabs obtained from the participants’ vagina, cervix, and endometrium were processed to isolate total DNA and amplify and sequence a portion from V3–V4 region of bacterial 16S rRNA. We recorded a total of 6,664,725 amplicon sequences (ASs), ranging between 401 and 100,970 ASs per sample (average 35.078). Distinct amplicon sequence variants (ASVs) (*n* = 1,487) were merged into 703 centroids based on pairwise similarity (threshold identity 0.97) clustering and 160 ASVs were removed with Barrnap, 543 ASVs were retained. Of these ASVs, three were removed because the taxonomic string contained any of (mitochondria, chloroplast) or had fewer than two total read counts over all samples. Finally, 540 unique ASVs, from 63 vaginal, 62 cervical, and 55 endometrial swab samples entered taxonomical sequence classification. Nine endometrial swabs (six adenomyosis patients and three controls) were unable to be obtained due to either patient withdrawal from the procedure or failed sampling due to anatomic impedances due to adenomyosis.

With replicates, this amounted to 37,300 ASs per sample at median (min: 2,803, p25: 23,553, p75: 48,617, max: 100,968) and 101,602 ASs per patient at median (min: 30,098, p25: 79,560, p75: 127,942, max: 190,933). Per sample read and ASs counts are available in Table S1.

Alpha-diversity rarefaction analysis was performed and the sample rarefaction curves showed good saturation with increasing sequencing depth (Fig. S2). This means that the samples were sequenced at adequate depth and sequencing deeper would not have increased ASV diversity.

Microbiome composition did not change along the female reproductive tract, but the microbiomes of participants with adenomyosis were notably different from those in healthy controls.

**Fig 1:**
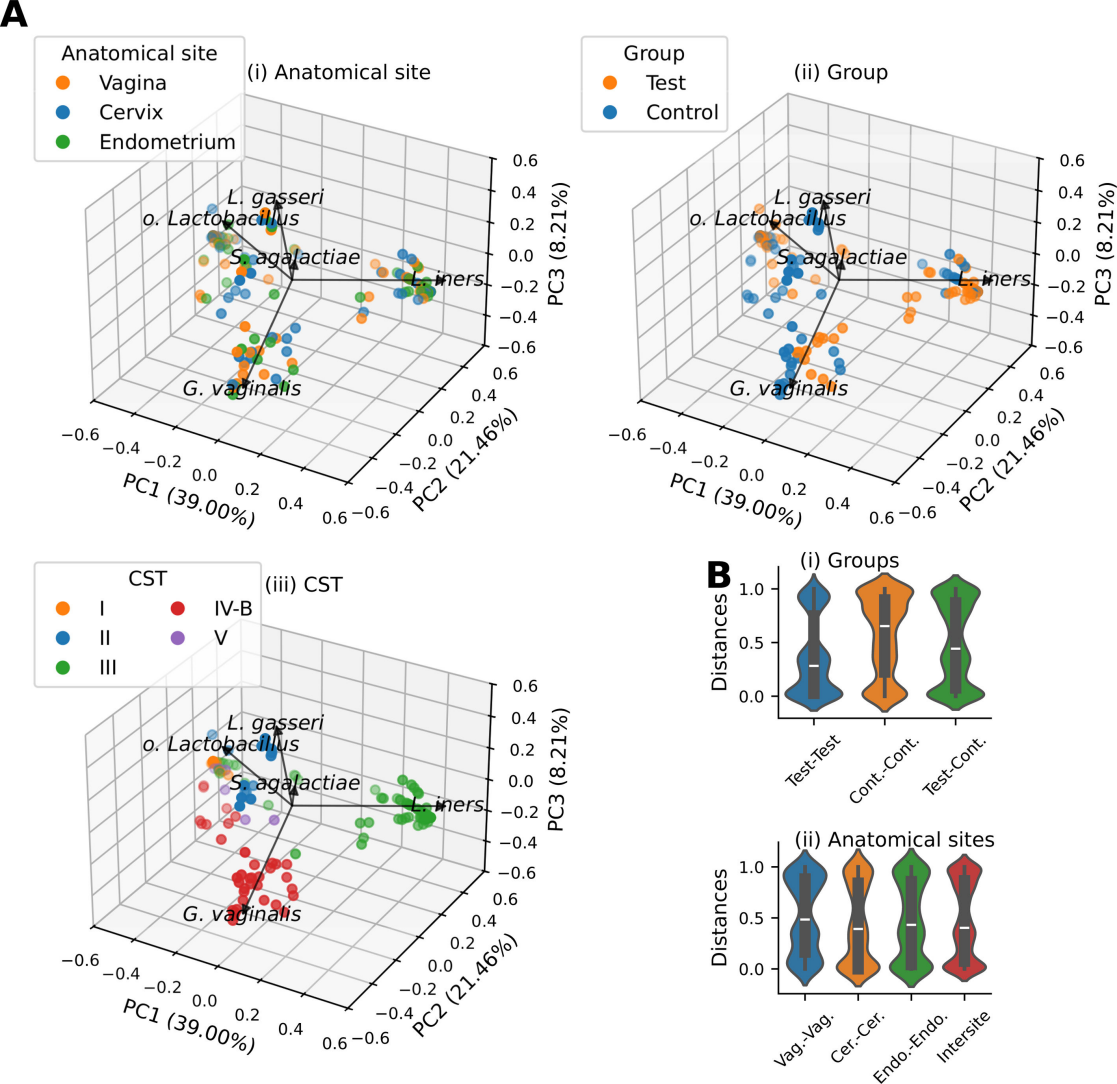
Microbiome beta diversity. (**A**) Principal coordinate (PC) projections of sample taxonomical relative abundance vectors into three dimensions, based on Bray-Curtis distances between relative abundance vectors at the taxonomical levels of species. Samples are colored according to anatomical site (i), experimental group (ii), and CST (iii). Loadings of the five most important taxa are shown as black vectors and illustrate their relative importance and direction in which they displace sample data. The most bacterial species driving data set variance were *L. iners, G. vaginalis, L. gasseri,* and sequences classified as *Lactobacillus* at the level of genera, but with unknown species-level assignment (*o. Lactobacillus*. Percentages of total variance explained by each PC are annotated along-side axis labels. (**B**) Beta diversity. Box-and-whiskers (box) and violin plots show intra- and intergroup Bray-Curtis distances between samples in specified grouping ([i] experimental group [Cont. = control] and [ii] anatomical site [Vag. = vagina, Cer. = cervix, Endo. = endometrium]). Violins show kernel-density-based distribution estimates. Medians and Q1–Q3 are shown with median mark and box, respectively; whiskers are drawn to the farthest datum from the box limit within 1.5 ×IQR.

Compositions of bacterial microbiota from the three different anatomical sites of the female RT, between study groups and between data stratified according to other covariates (listed in Table 1), were compared with each other by calculating Bray-Curtis distances between the taxonomical composition relative abundance vectors. Projection to three-dimensional coordinate space using principal coordinate analysis (PCoA) showed three diffuse groups of samples, suggesting three populations with distinct microbial compositions, governed by relative abundances of either *Lactobacillus iners, Gardnerella vaginalis,* or other lactobacilli (including *Lactobacillus gasseri* (Fig. 1A). Each cluster was formed of samples from all three anatomical locations thoroughly intermixed (Fig. 1Ai), suggesting no clear differences in bacterial composition of the sampled anatomical sites. On the other hand, we observed a weak tendency to separation of samples from women with adenomyosis and from healthy controls (Fig. 1Aii), with a larger proportion of adenomyosis samples in the cluster dominated by *L. iners*. Prediction of vaginal community state types showed that the microbiome profiles in the samples corresponded well to the canonical vaginal community state types (CSTs) (Fig. 1Aiii). Our cohort included CSTs I, II, III, IV-B, and V, which are typically dominated by *L. crispatus, L. gasseri, L. iners, G. vaginalis,* and *L. jensenii,* respectively (30). The *L. iners* dominated samples, corresponding with CST III, appeared to most frequently include adenomyosis samples. Coloring based on dysmenorrhea, chronic endometritis, heavy menstrual bleeding (HMB), and CPP mostly corresponded to subpopulations of the adenomyosis group.

The multivariate bacterial composition vectors grouped according to categorical attributes were compared with each other. Beta diversity box plots show different intra- and inter-group beta diversity distributions, but similar inter- and intra-site beta diversity distributions (Fig. 1B). Multivariate composition vector comparison results (PERMANOVA or ANOSIM, depending on PERMDISP test result) were consistently negative for comparisons of taxonomical composition vectors at classification levels of phylum, family, genus, and species (Table S2). However, we observed notable differences in microbiome compositions of participants with and without adenomyosis. The relevant test results were consistently positive at all four levels of taxonomical assignment and consistently at overall, and within each of the three anatomical sites.

*L. iners, L. gasseri,* and *G. vaginalis* are three major components of reproductive tract bacterial microbiota. The dominant taxonomical genus in the female RT was *Lactobacillus (Firmicutes, Lactobacilliaceae*), with 77.50% mean relative abundance (RA), followed by *Gardnerella (Actinomycetota, Bifidobacteriaceae*), with 13.90% mean RA, *Prevotella (Bacteriodota, Prevotellaceae*) at 1.30% mean RA, and *Streptococcus (Firmicutes, Streptococcaceae*) at 1.04% mean RA. All other genera fell below 1% at overall mean RA.

**Fig 2:**
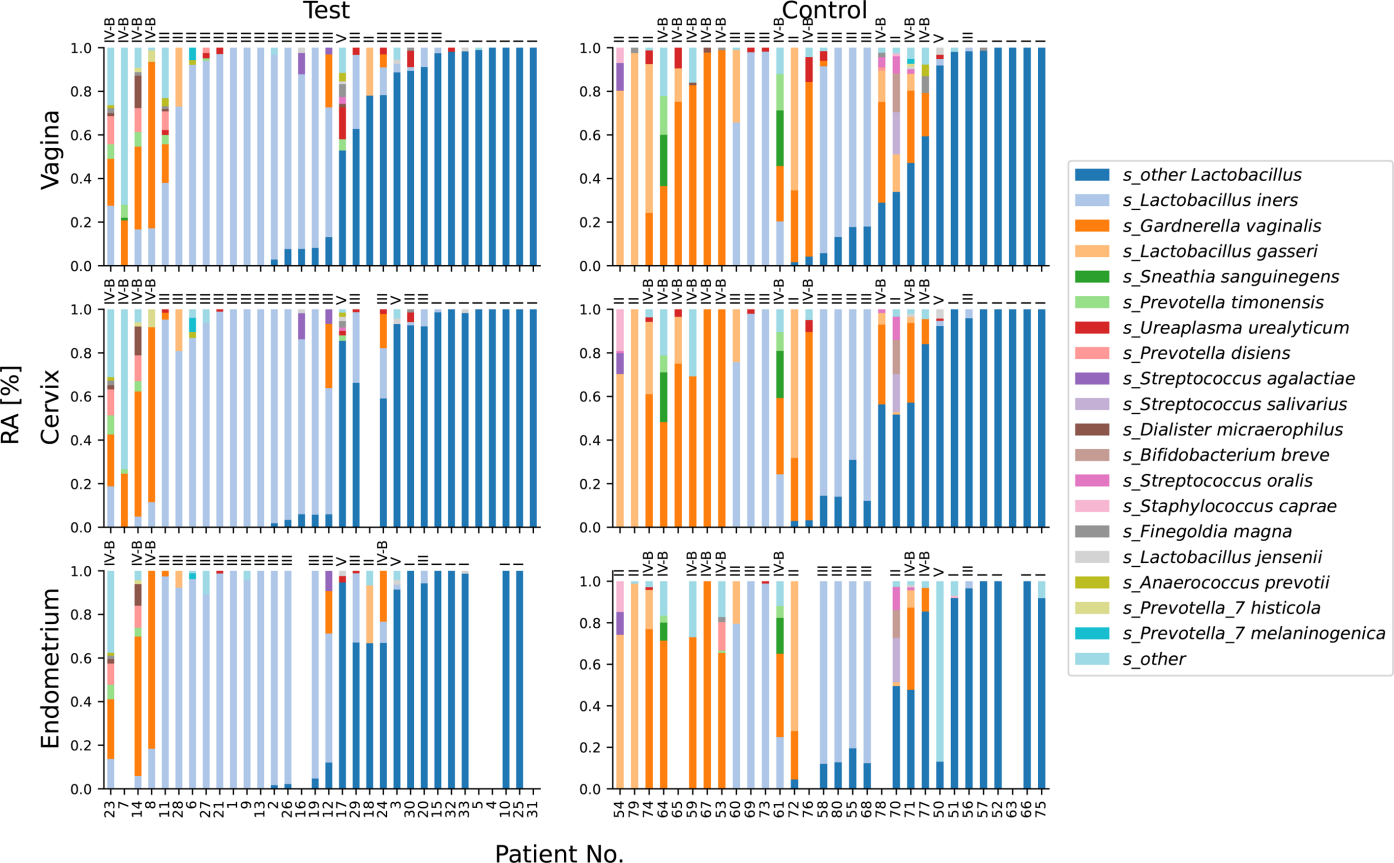
Bacterial compositions of samples. Stacked bar plots show relative abundances of bacterial species (color coded) in samples grouped according to anatomical site (rows) and experimental group (columns). Patient samples are ordered according to taxon abundances in the vagina; the same ordering of patients applies to samples from all three anatomical locations sampled. Each sample is annotated with the predicted CST (on top of each bar) as classified using VALENCIA software (30). Only the top 19 overall most abundant taxa are shown, all other taxa are collectively shown as “s_other.” Due to high conservation of the partial V3–V4 region of 16S rRNA amplified in the used assay, some *Lactobacillus* species are indistinguishable from each other (31, 32)*. Lactobacillus* species that could not be characterized to the species level are collectively reported as “*s_*other *Lactobacillus.”*

Detailed inspection showed that most samples in our cohort were dominated by *Lactobacillus,* most notably either by *L. gasseri, L. iners,* or the pseudo species level taxon “other *Lactobacillus*”—these are lactobacilli that could not be characterized to the species level. These were followed in much fewer samples dominated by *Gardnerella* (Fig. 2). Bacteria belonging to other genera in our cohort only appeared in sporadic samples, and in no case achieved dominance. Descriptive statistics of all identified genera, including counts, means, standard deviations, and percentile distributions, is available in Tables S3 and S4.

While amplicon sequencing of partial V3–V4 16S rRNA region is often sufficient to confidently characterize bacterial populations to the species level, some species, including some belonging to the *Lactobacillus* genus, for example *L. acidophilus/casei/crispatus/gallinarum,* cannot be distinguished from each other due to high sequence conservation (31, 32). We further used nearest centroid classification to predict which canonical microbial CSTs (30) are represented by our samples, which uses complete microbial profiles, not only species-level taxa. This analysis predicted multiple of our “other *Lactobacillus”* samples as CSTs III and I, which indicates more *L. iners* and some *L. crispatus* dominated samples, respectively (Fig. 2).

There were more *L. iners*-dominated samples among adenomyosis patients than in healthy individuals; *L. gasseri* and *G. vaginalis* dominated fewer adenomyosis microbiomes than those of healthy individuals.

We used ANCOM-BC analysis (33) to pinpoint specific microbial taxa with different RA’s in samples from different anatomical sites and study groups. ANCOM-BC was followed by careful inspection of bacterial compositions in Fig. 2 and RA distributions. Complete ANCOM-BC test results are available in Table S5. This testing indicated five bacterial species with different abundance levels in patients with adenomyosis and healthy controls. *G. vaginalis*, *L. gasseri* were negatively correlated with adenomyosis (negative W statistic), and *L. iners, A. prevotii, P. grossensis,* and *P. anaerobius* were positively (positive W statistic) correlated with adenomyosis. Indeed, Fig. 2 showed fewer *G. vaginalis* and *L. gasseri* dominant and more *L. iners* dominant samples in the adenomyosis group, with rather similar prevalences of samples dominated with lactobacilli of undetermined species. *A. prevotii, P. grossensis,* and *P. anaerobius* appeared only in a few samples and at very low RAs – they were not among the top 19 most abundant bacteria and were not included in Fig. 2, see Table S2 for metadata and S4 for details on specific RAs. *A. prevotii* appeared in eight adenomyosis samples with RAs 1.3%–3.8% and in one control sample with RA 5.3%. *P. grossensis* and P. *anaerobius* appeared in five adenomyosis samples each with RAs between 1.1%–3.0% and 1.3%–3.8%, respectively; the latter two bacteria did not appear in any samples from healthy individuals.

### Adenomyosis is associated with absence of *G. vaginalis* and *L. gasseri*, and with presence of *L. iners*

We evaluated presence/absence of specific species-level taxa as RA > 10% and used Fisher’s exact test or χ test to establish epidemiological association with anatomical site, study group, and other covariate attributes. This analysis associated adenomyosis with presence of *L. iners* (OR = 3.1) and absence of *L. gasseri* (OR = 0.3) and *G. vaginalis* (OR = 0) with adenomyosis. These were found to have different relative abundance distributions between study and control samples in ANCOM-BC; however, association testing further highlighted the absence of *Sneathia sanguinegens* in adenomyosis samples (OR = 0.14).

χ testing revealed significant association with adenomyosis between CST III and negative association with CSTs III and IV-B. Interestingly, according to CST prediction, several samples, appearing as dominated by lactobacilli of unknown species, showed as CST I, typically dominated by *L. crispatus* (Fig. 2); however, they had approximately the same prevalences in both groups and suggested no association.

Microbiome alterations correspond to distinct clinical features of adenomyosis. Associations of taxa presence/absence in relation to specific clinical symptoms of patients with adenomyosis were also detected. *L. iners* and *P. grossiensis* were positively associated with HMB, W = 7,062 and W = 2,993, respectively. Presence of lactobacilli other than *L. iners*, *L. gasseri,* and *L. jensenii* (other *Lactobacillus*) was associated with absence of HMB (W = −3,202). Dysmenorrhea was positively associated with *L. iners* (W = 5,731) and other species: *D. micraerophilus* (W = 3,289), *P. grossiensis* (W = 2,815*), P. timonensis* (W = 3,070), and *P. disiens* (W = 3,702), in contrast *L. gasseri and L. jensenii* showed negative association with dysmenorrhea (W= −5,465 and −2,882, respectively). *L. iners* was also associated with CPP (W = 4,352), whereas *L. gasseri* and *L. jensenii* were negatively associated with CPP at W= −6,073 and −3,686, respectively.

## DISCUSSION

In this study, we compared vaginal, cervical, and uterine microbiota of 33 patients with adenomyosis and of 31 healthy controls. The samples were obtained transcervically, in an office-based setting, from patients in reproductive age (mean age for both groups was 34.8 years). Results of our study showed no notable difference between vaginal, cervical, and endometrial bacterial microbiomes. The assessment of vaginal bacterial microbiome can thus reliably predict the state of cervical and endometrial microbiome. Female RT microbiomes outlined four clusters of patients with bacterial microbiota dominated either by *L. iners*, *L. gasseri*, and other lactobacilli, or *G. vaginalis*, which corresponded well with the canonical vaginal CSTs. Although all three clusters included both healthy women and adenomyosis patients, the RT microbiota composition in patients with adenomyosis differed from that of matched healthy controls. Adenomyosis microbiomes showed higher incidence of *L. iners*-dominant samples (CST III) were more common among adenomyosis samples than in samples from healthy individuals*,* and *L. gasseri* (CST II) and *G. vaginalis* (CST IV-B) more commonly dominated healthy microbiomes than those from patients with adenomyosis. Interestingly, even though we could not characterize any sequences to the level of *L. crispatus* species, VALENCIA software (30) predicted several samples as CST I, which are *L. crispatus* dominated. This was likely due to the software using nearest centroid classification which looks at statistical distances of samples from reference profiles of CSTs, which are constituted by RAs of higher taxonomical features, genera, and orders, instead of only species profiles. *A. prevotii*, *P. grossensis,* and *P. anaerobius* also appeared to weakly correlate with adenomyosis status. According to ANCOM-BC, some clinical symptoms of adenomyosis were associated with specific bacterial taxa; dysmenorrhea, HMB, and CPP were associated with *L. iners.* Additionally, *P. grossensis, P. disiens, P. timonesis,* and *D. micraerophilus* were associated with dysmenorrhea, and *P. grossensis* was associated with HMB. *L. gasseri* and *L. jensenii* appeared to anticorrelate with these symptoms.

Our results indicated no substantial differences between microbiome compositions at the three analyzed anatomical sites of the female RT. This may not be surprising as the three sites are anatomically and physiologically linked by cyclical reproductive processes. Disruptions of vaginal microbiota could therefore be propagated as far as the endometrial cavity. The composition of endometrial microbiota and its role remains uncertain and highly debated as it is considered a direct marker of uterine health. Our results show that these three sites form a continuum rather than distinct sites in terms of bacterial microbiota. This means that obtaining vaginal swabs, a simple and non-invasive procedure, may be sufficient for bacterial microbiome analysis or RT dysbiosis characterization.

Multivariate (beta diversity) analysis showed notable differences in RT microbiome compositions of women with adenomyosis in comparison with healthy controls. The differences appeared at the level of genera and species and were consistent in all three anatomical sites at the level of species. Previous studies investigating samples of patients with and without adenomyosis showed beta diversity differences in vaginal (23), endometrial (29, 34), and vaginal but not endometrial bacterial microbiomes (22). Collectively with this research corpus, our results favor the idea that disruptions of the reproductive microbiota are associated with adenomyosis and its clinical presentation (35). Our results are in line with those obtained in the largest previous studies of adenomyosis RT microbiome (22 [*N* = 38]), with only two larger studies in Chinese population, analyzing exclusively vaginal microbiome (23 [*N* = 47] and 36 [*N* = 40]).

This study consistently showed an association between adenomyosis and its clinical symptoms, and *L. iners* presence and high relative abundance. *L. iners* is a controversial species of *Lactobacillus*; it has often been reported in association with RT microbiota instability and appears to have an unfavorable role in fertility (37), implantation failure in IVF cycles (33, 38), and pregnancy loss (34, 39). Recent evidence suggests that *L. iners* is a transitional species that commonly colonizes the vagina after environmental disturbances such as BV and is less protective against vaginal dysbiosis than other lactobacilli (40, 41). Many studies recognized *L. iners* as a transitional and clinically ambivalent species, frequently associated with BV, associated with higher risk of relapse after BV treatment and heightened susceptibility to sexually transmitted infections (42).

*L. iners* has the smallest genome among the lactobacilli*,* and the ability of adaptation to both high and low pH environments (35, 43). Contrary to *L. crispatus*, *L. gasseri*, and *L. jensenii,* which can produce both D- and L-lactic acid by fermentation, *L. iners* lacks the gene for D-lactate dehydrogenase (*LDHD*) and can only produce L-lactic acid (44). L-lactate is an important metabolic fuel and intermediary (45–47), and a key signaling molecule, involved in metabolic programming, inflammation, transcriptional regulation through histone lactylation and chromatin remodeling 2 (48–51). The physiological role of D-lactate, on the other hand, is poorly understood. Unlike *L. iners*, humans encode both L- and D-lactate dehydrogenases (hLDHL, hLDHD). The two enzymes are from different protein families, and hLDHD (52) is expressed at an order of magnitude lower levels than hLDHL in nearly all surveyed tissues according to the Genotype-Tissue Expression Portal (53). The general conception is that D-lactate is a toxic metabolite, and D-lactic acidosis has been reported in association with gastric disorders, such as short bowel syndrome (SBS) and inflammatory bowel disease (54), and it has been reported to have a greater inhibitory effect on exogenous bacteria than L-lactic acid (55). Furthermore, a low ratio between L- and D-lactic acid concentration in feces has been suggested as a marker of microbiota disbalance regarding several conditions, such as SBS (56). The elevated L/D lactic acid ratio in the vagina may facilitate extracellular breakdown via extracellular matrix metalloproteinase inducer and matrix metalloproteinase-8 activation, which could help bacteria transverse the cervix and promote upper genital tract infections (57). Concentration of L-lactic acid, but not D-lactic acid, and the L/D-lactic acid ratio were significantly elevated in women with high-grade squamous cell intraepithelial lesion of the cervix and cervical cancer (58). *L. iners* found in cervical cancer was associated with decreased recurrence-free and overall survival in cervical cancer patients (59, 60). *L. iners* also lacks the ability to produce H_2_O_2_ through pyruvate oxidation (44, 61); it expresses a putative virulence factor (inerolysin), a cholesterol-dependent cytolysin that creates aqueous pores within the cell membrane, which may be one of the essential features required to obtain nutrients from the host (44, 62). Moreover, *L. iners* lacks transport mechanisms for cysteine and cysteine-containing molecules (63). In vaginal epithelial cells, *L. iners* can upregulate the pattern-recognition receptor signaling pathway and increase the expression of tumor necrosis factor (43, 64). Some studies suggest that *L. iners* can activate the toll-like receptor signaling pathway, increase heat shock protein 70 expression, and inhibit autophagy, which could destroy the local homeostasis and reduce the ability of vaginal epithelial cells to recognize and respond to potential pathogens (64, 65).

Although the exact role of *L. iners* in adenomyosis remains unknown, finding this association does point towards therapeutic options to improve clinical symptoms and reproductive outcomes. Probiotic treatment has shown beneficial results in various gynecological conditions associated with microbiome disruption, such as vaginal infections, polycystic ovary syndrome (66), and endometriosis (67), but future studies regarding probiotic supplementation in adenomyosis are needed. Not many other therapeutic strategies for *L. iners* reduction have previously been proposed; one of them being supplementation of cystine uptake inhibitors, where it has been demonstrated that an inhibitor with metronidazole promotes *L. crispatus* dominance, that is a marker of vaginal health (63). Supplementation of D-lactate dehydrogenase or D-lactate producing bacteria to lower L/D lactic acid ratio could be beneficial in patients with adenomyosis. However, *hLDHD* mutations leading to deficient activity have been found in patients showing D-lactoacidosis (52). Based on our results, it remains unclear whether *L. iners* was the cause or herald of the clinical symptoms or adenomyosis. Unlike in the gastrointestinal system, where it can be of alimentary origin, in the female RT, lactate can be either of physiological or bacterial origin. A deficient hLDHD may lead to reduced physiological D-lactate concentration. Given *L. iners’* lack of *LDHD*, the resulting low D-lactate environment may be a more opportune environment for its overgrowth. In this case, excessive supplementation with D-lactate, LDHD, or LDHD-expressing lactobacilli might cause symptom aggravation, D-lactoacidosis, or hyperuricemia (68, 69). LDHL supplementation has been debated in cancer therapy, together with other possible therapeutic mechanisms; application of bacteriocins, lytic phages, bioengineered bacteria, or clinically proven probiotics (59). The knowledge about *L. iners* and the fact that clinically available LDH inhibitors already exist could possibly benefit treatment of adenomyosis.

On the other hand, a potential positive role of *L. iners* has recently been proposed (70), suggesting it appears in association with BV and gynecological issues due to its ability to persist under unstable conditions. This finding was reported in pregnant women during the third trimester, a period associated with a physiologically induced mild pro-inflammatory environment that may promote an environment favorable for *L. iners* proliferation. Its metabolic plasticity likely underpins its ability to coexist with BV-associated taxa without conferring protective effects and maintaining a microbial equilibrium during this stage of pregnancy. As pregnancy is a very specific physiological state that makes it difficult to extend this finding to other contexts. Given the considerable genetic diversity among *L. iners* strains, strain-level analyses would be needed, critical for more accurately assessing their impact on health outcomes.

An interesting finding of our study was that *G. vaginalis* was one of the three components dominating female RT microbiomes, and in contrast to previous research, presence of *G. vaginalis* was negatively associated with adenomyosis and its clinical symptoms (23). *G. vaginalis* is a predominantly anaerobic bacterium, traditionally considered pathogenic and associated with bacterial vaginosis (63, 71). During pregnancy, high-abundance *G. vaginalis* is reportedly associated with adverse pregnancy outcomes such as preterm birth, premature rupture of membranes, and intra-amniotic infection (72). According to the literature, in the non-pregnant population, up to 50% of women with *G. vaginalis* do not develop symptoms (64, 65, 73, 74). However, in our study, with 31 out of 48 samples from asymptomatic women, this proportion was even higher, 64.6% (CI95%: 51.1%–78.1%). Recently, whole genome sequencing and advanced molecular approaches (e.g., chaperonin 60 sequencing) have enabled the identification of several *Gardnerella* genomospecies (*G. vaginalis, G. swidsinskii, G. leopoldii, G. piotii*), with different virulence and ecological behavior depending on sialidase activity, biofilm formation ability, etc. (75–77). *G. vaginalis* is therefore a member of a genomically diverse group, in which some strains are virulent, some are commensal, and evidence to support different genomospecies to BV is often inconclusive (76). As 16S rRNA gene sequencing does not detect differences between the genomospecies, further genomic and functional profiling will be key to delineate *G. vaginalis'* contribution to healthy or BV pathophysiology.

Besides its pronounced association with *L. iners*, we also observed low-grade correlations with adenomyosis and *A. prevotii*, *P. grossensis*, and *P. anaerobius*. They are all low-abundance Gram-positive anaerobic cocci (GPAC) whose clinical significance in adenomyosis remains uncertain. *P. anaerobius*, a commensal of the oral and gastrointestinal microbiota with reported associations to colorectal carcinogenesis (78), is rarely detected in healthy vaginal microbiota. Emerging evidence suggests that this organism may promote inflammation and contribute to oncogenic processes in the cervix, including enhanced angiogenesis, although these findings require further validation (79, 80). *A. prevotii* is an opportunistic anaerobe occasionally identified in low abundance in the female RT in association with dysbiosis. Although often part of the normal flora of the skin, oral cavity, and gut, it may act as an opportunistic pathogen, particularly in polymicrobial infections or compromised hosts (81). *P. grossensis* is a GPAC that is very rarely implicated in human disease (82).

We additionally found differences in relative taxa abundances in relation to clinical symptoms of patients with adenomyosis. In accordance with our results*,* genus *Prevotella* has been proposed as a possible adenomyosis biomarker by Lin et al. (34). Research shows that *Prevotella* species are commonly detected in women with diverse anaerobic vaginal bacterial composition with or without BV (83), and have been associated with increased risk for acquisition of sexually transmitted infections, including HIV-1 (84), preterm birth, and premature rupture of membranes (85–87). As colonizers of gut and oral microbiome, *Prevotella* spp. are associated with autoimmune diseases like rheumatoid arthritis, type 2 diabetes, obesity, intestinal dysbiosis, and periodontitis (87–89). *Prevotella disiens* has been detected in endometrial or fallopian tube samples collected from women with BV (88), pelvic inflammatory disease (90), and pyometra (85). *P. disiens* exhibits high cytotoxic activity, with possible ammonia production in epithelia, confirming that it has proinflammatory potential and potential to damage endometrial epithelial cells (88, 91, 92). *P. timonensis* is a characteristic member of vaginal dysbiosis that has distinct virulence-related properties that include initial adhesion and a high capacity for mucin degradation at the vaginal epithelial mucosal surface (93) and is known to induce a strong proinflammatory response through dendritic cell activation (94). *D. micraerophilus* is an obligate anaerobic gram-negative bacillus, possibly associated with gynecological infections and has been described as a cause of bacteremia in pyometra (95). Not much is known about their possible role in vaginal, cervical, or endometrial dysbiosis. Given the modest correlation and limited evidence, the contribution of these bacterial species to adenomyosis cannot yet be defined, and further studies are needed to elucidate their potential contributions to disease mechanisms.

### Strengths and limitations

#### Strengths

This is the first comprehensive analysis of vaginal, cervical, and endometrial samples obtained using a minimally invasive, transcervical approach in a cohort of Caucasian women during the window of implantation, with a high degree of statistical validity and reliability. It revealed that the least invasive option, vaginal microbiome sampling, reliably predicts the microbiome composition of cervix as well as endometrium. *L. iners* could be a good indicator of microbiome instability and susceptibility to infection or inflammation, and here we propose different therapeutic strategies of microbiota remodeling that have not yet been proposed in the treatment of adenomyosis. Furthermore, our findings support previous calls by researchers for a context-dependent interpretation of *G. vaginalis* colonization. We show that it might be an important dominant component of RT microbiota, similar to what is perceived for non-*L*. *iners* lactobacilli.

#### Limitations

In microbiome sequencing analyses, contamination can potentially lead to erroneous results. Two types of contamination could be anticipated in our case. First, biomass carryover contamination might have occurred during sampling in the clinical setting: to obtain cervical swabs, the clinician had to pass the vagina and both the vagina and cervix to obtain endometrial swabs. Due to its low biomass, the uterine cavity is especially prone to carryover microbiota from the lower two RT sites. In our study, we attempted to mitigate carryover contamination with careful sampling through telescopic catheters spanning the lower sites, as described in Materials and Methods. Transcervical sampling approach is a minimally invasive, office-based method of obtaining uterine microbiota that has been shown to yield results comparable to those obtained through invasive surgical sampling (8). A recent study has identified a double-sheathed catheter approach—methodologically similar to ours—as an optimal uterine microbiome sampling method (96). Our results showed no difference in microbial composition between different anatomical sites, which may have been due to this type of contamination. This result could, however, reflect the physiological continuity of RT in women of reproductive age, in which the uterine cavity remains in a dynamic interaction with the lower RT. Physiological and cyclical changes contribute to the inherent difficulty of defining a distinct endometrial microbiota. Importantly, our findings, in line with previous studies (97), suggest that the vaginal microbiome may reflect the uterine microenvironment. To date, only a single study—conducted in reproductive-age women with adenomyosis and employing transcervical sampling of the uterine microbiota—has been published, and its findings were consistent with ours (22). In our study, lactobacilli were identified as the predominant colonizers of the uterine cavity in both patient groups, a finding that is further supported by previous reports (16, 98, 99).

Second, the samples could have been cross-contaminated at any point during laboratory handling. Again, the most vulnerable would have been the endometrial samples due to low expected biomass. Sample cross-contamination was mitigated by careful handling: all samples were processed by skilled operators, in aseptic conditions with laminar airflow, and handling different samples was decoupled in space-time: two vials of different samples were never opened at the same time at the same workbench. By implementing meticulous sampling procedures and adhering to rigorous laboratory standards—including the systematic use of positive and negative PCR controls—we substantially reduced the risk of contamination originating from our workflow. One possible approach would be the use of so-called blank controls (100), in which endometrial samples are intentionally contaminated and compared with the remaining samples. However, in our context—where we study patients with adenomyosis and healthy participants—it is already a considerable challenge to recruit women willing to undergo sampling of the uterine cavity, making such an approach difficult to implement. This would expose participants to unnecessary risk and require discarding informative and valuable material.

We used the V3–V4 region of 16S rRNA gene for taxonomic assignment and microbiome composition analysis. While the 16S rRNA gene is a universal bacterial marker, it is present in variable copy numbers across different bacterial taxa (101), which may hinder accurate stoichiometric estimation of bacterial composition. Furthermore, due to limited species-level resolution of V3–V4 16S rRNA region, some sequences could not be assigned to species-level taxa. Due to this, we were unable to characterize *L. crispatus*, for example; however, we were to some degree able to mitigate this issue by analyzing CSTs, as CSTs typically dominated by *L. crispatus* could be determined. With the advances in long-read sequencing technologies (e.g., PacBio and Oxford Nanopore) (102), future studies may mitigate this problem by sequencing the entirety of the 16S rRNA gene or by choosing a different pan-proteobacterial gene with lower copy-number variation such as *rpoB* (103, 104). Additionally, mock bacterial communities could be used as a control to account for possible contaminations or errors during the entirety of the sequencing process (105).

Microbiota analysis could theoretically produce predictors and diagnostic tools for non-specific symptoms like dysmenorrhea, CPP, and HMB. In this aspect, the study could be supplemented by more thorough evaluation of clinical symptoms of patients to show symptom range and heterogeneity in correlation with microbial compositions. The heterogeneous range of results of previous studies may be a consequence of different patient populations (geographic, ethnic groups), obtaining of samples (surgical approaches vs. outpatient visits), vaginal disinfection prior to endometrial sampling, and unstandardized microbiome analysis.

Finally, although this is one of the largest analyses of microbiome in adenomyosis, inclusion of larger sample and patient cohorts could reinforce our findings in future studies.

## MATERIALS AND METHODS

### Participant inclusion

Our observational cross-sectional study group included 33 women with clinically and ultrasonographically confirmed adenomyosis as experimental group and 31 healthy controls. All enrolled women were informed in detail about the potential risks associated with sample collection and the purpose of the study and voluntarily signed written consent to participate in the study.

Participant inclusion and exclusion criteria are shown in Table 2. Adenomyosis was diagnosed according to the following criteria established by the Morphological Uterus Sonographic Assessment (MUSA) group (106, 107). Ultrasound diagnostics of adenomyosis was performed by experienced sonographers in the Tertiary Referral Center for Endometriosis Care, Department of Human Reproduction, Division of Obstetrics and Gynecology, University Clinical Hospital, Ljubljana, Slovenia. We performed an internal evaluation of the past ultrasonographic predictive value of adenomyosis for two sonographers (a correlation of ultrasound and histologic confirmation of diagnosis of adenomyosis), and it was 96% separately for both sonographers. We collected clinical and sociodemographic data of the participants prior to or during the visit; age, body mass index, nulliparity, number of deliveries and spontaneous miscarriages, infertility, and infertility treatment. Patients with adenomyosis were evaluated for adenomyosis-typical subjective symptoms (dysmenorrhea, CPP defined by pain in the lower abdomen or pelvis lasting for more than 6 months [108] and HMB). The control group included age-matched women with other non-inflammatory gynecological conditions who were treated at the same institution as participating patients with adenomyosis. All samples were collected between October 2020 and April 2023.

**TABLE 2:** Participant inclusion and exclusion criteria^*a*^

Inclusion criteria	Exclusion criteria
Study group: – age from 18 to 41 years – presence of ultrasound signs of adenomyosis according to the MUSA criteria: direct features such as presence of myometrial cysts, myometrial hyperechoic islands, echogenic subendometrial lines, and buds and indirect features such as diffusely enlarged or globular uterus, asymmetry of the uterine walls, fan shaped shadowing, translesional vascularity, irregular or interrupted junctional zone Control group: – 18- to 41-year-old asymptomatic women – treatment at the Department of Human Reproduction due to male factor infertility and patients planned for laparoscopic sterilization – no previous history of gynecological conditions	Both groups: – acute inflammation of the upper or lower genitals and urinary tract – antibiotic or antifungal treatment in the last 30 days – use of hormonal preparations and IUDs – known neoplasia, inflammatory or autoimmune disease – pregnancy or lactation – previous application of vaginal preparations (less than 10 days) – intrauterine procedures within 4 weeks (less than one menstrual cycle after the procedure)

^*a*^ MUSA, morphological uterus sonographic assessment; IUD, intrauterine device.

### Collection of samples

All samples were collected on the 22nd day of the menstrual cycle, which corresponds to the so-called window of implantation. Under aseptic conditions, vaginal, cervical, and endometrial swabs were obtained using Copan Regular Tip Flocked Swab with 2 mL eNAT Medium (Copan Diagnostics, Murrieta, CA, USA). The uterine cavity swabs were collected prior to ultrasound examination, following vaginal and cervical swab collection. An endometrial swab was obtained by inserting a sterile speculum and using a sterile sheath to pass through the cervix, thereby minimizing contamination from the vaginal or cervical microbiota (demonstrated in Fig. S1). Subsequently, an endometrial sample was obtained for histopathological diagnostics using aspiration biopsy with an endometrial pipelle to confirm the corresponding menstrual phase. Samples were transported in liquid nitrogen and stored at –80°C. Endometrial samples were additionally subjected to histopathological examination and immunohistochemical staining (CD138/syndecan in plasma cells) to detect the presence of chronic endometritis.

### DNA extraction, 16S rRNA gene amplification, and sequencing

Genomic DNA from vaginal, cervical, and endometrial samples was extracted using EZ1 Virus Mini Kit V.2 (Qiagen, Hilden, Germany) and BioRobot EZ1 according to the manufacturer’s instructions. The extracted DNA was quantified using Invitrogen Qubit 4 Fluorometer (ThermoFisher, Waltham, MA, USA). All isolates were then subjected to real-time polymerase chain reaction (RT-PCR) in order to determine the concentration and quality of DNA, based on the amplification of the 150 bp of the human beta-globin gene on the LightCycler 2 (Roche Diagnostics, Mannheim, Germany) followed by 16S rRNA gene amplification by RT PCR (Molzym COMPLETE RT-PCR MMX QuantiTect Probe PCR + UNG Kit, Qiagen) according to the manufacturer’s instructions on the LightCycler 480 (Roche Diagnostics). DNA in our samples was in sufficient amount and quality for successful 16S rRNA gene amplification (with CT values ranging from 12,33 to 26,09). V3–V4 regions on the 16S rRNA were amplified with specific primers and sequencing libraries were prepared according to the Illumina 16S Metagenomic Sequencing Library Preparation protocol (Illumina MiSeq System, San Diego, CA, USA). In brief, full-length primer sequences (in standard IUPAC nucleotide nomenclature) targeting this region were 16S Amplicon PCR Forward Primer (5′ TCGTCGGCAGCGTCAGATGTGTATAAGAGACAGCCTACGGGNGGCWGCAG) and 16S Amplicon PCR Reverse Primer (5′ GTCTCGTGGGCTCGGAGATGTGTATAAGAGACAGGACTACHVGGGTATCTAATCC).

16S rRNA PCR was carried out with KAPA HiFi HotStart ReadyMix (Roche Diagnostics) on MiniAmp Thermal Cycler (Thermo Fisher). The amplification was conducted at 95°C for 3 min, 25× the following: 95°C for 30 s, 55°C for 30 s, 72°C for 30 s and 1× 5 min at 72°C. PCR product sizes were verified using the 2100 Bioanalyzer and High Sensitivity DNA kit (Agilent, Santa Clara, CA, USA). Index PCR for sequencing library preparation was performed (IDT for Illumina DNA/RNA UD Indexes, Integrative DNA Technologies, USA), libraries were purified using AMPure XP beads (Beckman Coulter, Brea, CA, USA), and quantified using 2100 Bioanalyzer using High Sensitivity DNA kit to determine fragment lengths and Qubit Fluorometer using the Qubit dsDNA HS and BR Assay Kits (Thermo Fisher) to determine DNA concentrations. Combined sequencing libraries were denatured and diluted using 0.2 M NaOH and supplemented with HT1 hybridization buffer according to the manufacturer’s instructions (Illumina). Prior to sequencing, the libraries were combined with PhiX control at a ratio of 510 µL: 90 µL. MiSeq sequencing was performed using the MiSeq Reagent Kit v3 (Illumina). Sequencing generated a total of 11,972,080 read pairs.

### Statistical and bioinformatics analysis

The sequencing data set was processed using nf-core/ampliseq pipeline version 2.8.0 (109). Sequence read quality was evaluated using FastQC (110) and summarized with MultiQC (111). Cutadapt (112) was used to trim primers and all untrimmed sequences were removed; sequences that did not contain primer sequences were considered artifacts and were also removed. At mean, 78.9% of the sequence read pairs per sample passed the Cutadapt primer filtering. Trimmed sequenced read pairs were processed with DADA2 (113) to eliminate PhiX contamination, trim reads (before median quality drops below 25 and at least 75% of reads are retained; forward reads at 283 bp and reverse reads at 210 bp, reads shorter than this were discarded), discard reads with > 2 expected errors, correct errors, merge read pairs, and remove PCR chimeras. DADA2 was used to extract and filter ASVs, VSEARCH was used to merge the ASVs into centroids, and Barrnap was used to remove ASVs that did not correspond to bacterial or archaeal sequences.

Taxonomic classification was performed with DADA2 and the database ‘Silva 138.1 prokaryotic SSU’ (114). ASV sequences, abundance, and DADA2 taxonomic assignments were loaded into QIIME2 (115).

Absolute and relative abundance tables were further inspected by custom analyses post-pipeline completion. The bottom 5 percentiles of samples according to ASV counts were excluded from further analyses, and relative abundances in sample composition vectors below 1% were set to zero and the relative abundances were recomputed.

Analyses and visualization were carried out using custom Python 3 scripts; (relative) abundance vectors were represented as Numpy v1.24.3 (116) arrays, and Bray-Curtis distances were calculated using Scipy v1.13.1 (117). Data organization was facilitated with Pandas v1.5.3 (118). PCoAs were carried out using Scikit-bio v0.6.2 (119) and plotted using Matplotlib v3.7.2 (120) and Seaborn v0.12.2 (121).

The two study groups were compared with each other based on covariate categorical and continuous variables. Continuous variables were compared using the Mann-Whitney *U* test. Categorical variables were compared using Fisher’s exact test wherever possible (2 × 2 contingency tables), χ test was used in cases of larger contingency tables. Statistical analyses of covariates were performed using R Software v4.4.0 and functions from library Epitools.

Differences in beta diversity between groups were tested using PERMANOVA and ANOSIM tests as applicable, according to PERMDISP test results. ANCOM analysis was used to find specific compositional differences between groups, Mann-Whitney U, Kruskal-Wallis, and one-way ANOVA tests were used depending on number of groups involved and other test restrictions; Benjamini-Hochberg false discovery rate corrected *P*-value at threshold level 0.05 was used to determine test result. PERMDISP, PERMANOVA, ANOSIM, and ANCOM tests were performed using Python 3 scripts as implemented in Scikit-bio.

## Data Availability

All raw sequencing data have been deposited in the NCBI Sequence Read Archive under BioProject accession number PRJNA1373171.

## SUPPLEMENTAL MATERIAL

File 1 spectrum.02791-25-s0001.docx Data processing pipeline parameters.

Figure S1 spectrum.02791-25-s0002.docx Graphic representation of endometrial sampling.

Figure S2 spectrum.02791-25-s0003.docx Rarefaction curves.

Table S1 spectrum.02791-25-s0004.xlsx Clinical metadata and sequencing information of all included samples.

Table S2 spectrum.02791-25-s0005.xlsx Beta-diversity difference testing results.

Table S3 spectrum.02791-25-s0006.xlsx Descriptive statistics of relative abundance distributions of all detected bacterial species and genera (in sheets).

Table S4 spectrum.02791-25-s0007.xlsx Complete per-sample relative abundances of all detected bacterial species and genera (in sheets).

Table S5 spectrum.02791-25-s0008.xlsx Complete ANCOM-BC analysis results, including bias-corrected abundance estimates and statistical significance for all detected taxa.

ASM does not own the copyrights to Supplemental Material that may be linked to, or accessed through, an article. The authors have granted ASM a non-exclusive, world-wide license to publish the Supplemental Material files. Please contact the corresponding author directly for reuse.
