# Supplementary material for: Bacterial microbiome analysis of vaginal, cervical, and endometrial samples in patients with adenomyosis during the window of implantation
Source: Microbiol Spectr. 2026 Feb 18;14(4):e02791-25. doi: 10.1128/spectrum.02791-25 (PMC13055214; doi:10.1128/spectrum.02791-25)
Supplement: File 1 — Data processing pipeline parameters. [file spectrum.02791-25-s0001.docx]

|  |  |
| --- | --- |
| input | null |
| input_fasta | null |
| input_folder | "./data/" |
| extension | "/*_R{1,2}_001.fastq.gz" |
| pacbio | false |
| iontorrent | false |
| FW_primer | "CCTACGGGNGGCWGCAG" |
| RV_primer | "GACTACHVGGGTATCTAATCC" |
| classifier | null |
| metadata | "./metadata.tsv" |
| trunc_qmin | 25 |
| trunc_rmin | 0.75 |
| trunclenf | null |
| trunclenr | null |
| max_ee | 2 |
| max_len | null |
| ignore_failed_filtering | false |
| min_len | 50 |
| metadata_category | null |
| metadata_category_barplot | null |
| double_primer | true |
| retain_untrimmed | false |
| cutadapt_min_overlap | 3 |
| cutadapt_max_error_rate | 0.1 |
| exclude_taxa | "mitochondria,chloroplast" |
| min_frequency | 2 |
| min_samples | 1 |
| multiple_sequencing_runs | false |
| single_end | false |
| sample_inference | "independent" |
| illumina_novaseq | false |
| illumina_pe_its | false |
| concatenate_reads | false |
| cut_its | "none" |
| its_partial | 0 |
| picrust | true |
| sbdiexport | false |
| addsh | false |
| tax_agglom_min | 2 |
| tax_agglom_max | 6 |
| min_read_counts | 1 |
| ignore_failed_trimming | true |
| ignore_empty_input_files | false |
| qiime_adonis_formula | null |
| seed | 100 |
| filter_ssu | "bac,arc" |
| min_len_asv | null |
| max_len_asv | null |
| filter_codons | null |
| orf_start | 1 |
| orf_end | null |
| stop_codons | "TAA,TAG" |
| pplace_tree | null |
| pplace_aln | null |
| pplace_model | null |
| pplace_alnmethod | "hmmer" |
| pplace_taxonomy | null |
| pplace_name | null |
| diversity_rarefaction_depth | 500 |
| ancom_sample_min_count | 1 |
| vsearch_cluster | true |
| vsearch_cluster_id | 0.97 |
| report_template | "/root/.nextflow/assets/nf-core/ampliseq/assets/report_template.Rmd" |
| report_css | "/root/.nextflow/assets/nf-core/ampliseq/assets/nf-core_style.css" |
| report_logo | "/root/.nextflow/assets/nf-core/ampliseq/assets/nf-core-ampliseq_logo_light_long.png" |
| report_title | "Summary of analysis results" |
| report_abstract | null |
| skip_cutadapt | false |
| skip_dada_quality | false |
| skip_barrnap | false |
| skip_qiime | false |
| skip_qiime_downstream | false |
| skip_fastqc | false |
| skip_alpha_rarefaction | false |
| skip_abundance_tables | false |
| skip_barplot | false |
| skip_taxonomy | false |
| skip_dada_taxonomy | false |
| skip_dada_addspecies | false |
| skip_diversity_indices | false |
| skip_ancom | false |
| skip_multiqc | false |
| skip_report | false |
| dada_ref_taxonomy | "silva=138" |
| dada_assign_taxlevels | null |
| dada_ref_tax_custom | null |
| dada_ref_tax_custom_sp | null |
| cut_dada_ref_taxonomy | false |
| dada_addspecies_allowmultiple | false |
| dada_taxonomy_rc | true |
| sintax_ref_taxonomy | null |
| qiime_ref_taxonomy | null |
| qiime_ref_tax_custom | null |
| kraken2_ref_taxonomy | null |
| kraken2_assign_taxlevels | null |
| kraken2_ref_tax_custom | null |
| kraken2_confidence | 0 |
| multiqc_config | null |
| multiqc_title | null |
| multiqc_logo | null |
| max_multiqc_email_size | "25.MB" |
| multiqc_methods_description | null |
| outdir | "./20240516_1_rev20240520v5-silva=138_fcutoff2-out" |
| publish_dir_mode | "copy" |
| email | null |
| email_on_fail | null |
| plaintext_email | false |
| monochrome_logs | false |
| hook_url | null |
| help | false |
| version | false |
| config_profile_name | null |
| config_profile_description | null |
| custom_config_version | "master" |
| max_cpus | 100 |
| max_memory | "900.GB" |
| qiime2_ref_taxonomy | "silva=138" |
| custom_config_base | "<https://raw.githubusercontent.com/nf-core/configs/master>" |
| config_profile_contact | null |
| config_profile_url | null |
| max_time | "240.h" |
| validationFailUnrecognisedParams | false |
| validation-fail-unrecognised-params | false |
| validationLenientMode | false |
| validation-lenient-mode | false |
| validationSchemaIgnoreParams | "dada_ref_databases,qiime_ref_databases,sintax_ref_databases,kraken2_ref_databases,genomes,igenomes_base" |
| validation-schema-ignore-params | "dada_ref_databases,qiime_ref_databases,sintax_ref_databases,kraken2_ref_databases,genomes,igenomes_base" |
| validationShowHiddenParams | false |
| validation-show-hidden-params | false |
| validate_params | true |
| dada_ref_databases |  |
| coidb |  |
| title | "COIDB - CO1 Taxonomy Database - Release 221216" |
| file |  |
| 0 | "<https://figshare.scilifelab.se/ndownloader/files/38787072>" |
| 1 | "<https://figshare.scilifelab.se/ndownloader/files/38787069>" |
| citation | "Sundh J, Manoharan L, Iwaszkiewicz-Eggebrecht E, Miraldo A, Andersson A, Ronquist F. COI reference sequences from BOLD DB. doi: <https://doi.org/10.17044/scilifelab.20514192.v2>" |
| fmtscript | "taxref_reformat_coidb.sh" |
| dbversion | "COIDB 221216 (<https://doi.org/10.17044/scilifelab.20514192.v2>)" |
| coidb=221216 |  |
| title | "COIDB - CO1 Taxonomy Database - Release 221216" |
| file |  |
| 0 | "<https://figshare.scilifelab.se/ndownloader/files/38787072>" |
| 1 | "<https://figshare.scilifelab.se/ndownloader/files/38787069>" |
| citation | "Sundh J, Manoharan L, Iwaszkiewicz-Eggebrecht E, Miraldo A, Andersson A, Ronquist F. COI reference sequences from BOLD DB. doi: <https://doi.org/10.17044/scilifelab.20514192.v2>" |
| fmtscript | "taxref_reformat_coidb.sh" |
| dbversion | "COIDB 221216 (<https://doi.org/10.17044/scilifelab.20514192.v2>)" |
| gtdb |  |
| title | "GTDB - Genome Taxonomy Database - Release R08-RS214.1" |
| file |  |
| 0 | "<https://data.ace.uq.edu.au/public/gtdb/data/releases/release214/214.1/genomic_files_reps/bac120_ssu_reps_r214.tar.gz>" |
| 1 | "<https://data.ace.uq.edu.au/public/gtdb/data/releases/release214/214.1/genomic_files_reps/ar53_ssu_reps_r214.tar.gz>" |
| citation | "Parks DH, Chuvochina M, Waite DW, Rinke C, Skarshewski A, Chaumeil PA, Hugenholtz P. A standardized bacterial taxonomy based on genome phylogeny substantially revises the tree of life. Nat Biotechnol. 2018 Nov;36(10):996-1004. doi: 10.1038/nbt.4229. Epub 2018 Aug 27. PMID: 30148503." |
| fmtscript | "taxref_reformat_gtdb.sh" |
| dbversion | "GTDB R08-RS214.1 (<https://data.ace.uq.edu.au/public/gtdb/data/releases/release214/214.1>)" |
| gtdb=R08-RS214 |  |
| title | "GTDB - Genome Taxonomy Database - Release R08-RS214.1" |
| file |  |
| 0 | "<https://data.ace.uq.edu.au/public/gtdb/data/releases/release214/214.1/genomic_files_reps/bac120_ssu_reps_r214.tar.gz>" |
| 1 | "<https://data.ace.uq.edu.au/public/gtdb/data/releases/release214/214.1/genomic_files_reps/ar53_ssu_reps_r214.tar.gz>" |
| citation | "Parks DH, Chuvochina M, Waite DW, Rinke C, Skarshewski A, Chaumeil PA, Hugenholtz P. A standardized bacterial taxonomy based on genome phylogeny substantially revises the tree of life. Nat Biotechnol. 2018 Nov;36(10):996-1004. doi: 10.1038/nbt.4229. Epub 2018 Aug 27. PMID: 30148503." |
| fmtscript | "taxref_reformat_gtdb.sh" |
| dbversion | "GTDB R08-RS214.1 (<https://data.ace.uq.edu.au/public/gtdb/data/releases/release214/214.1>)" |
| gtdb=R07-RS207 |  |
| title | "GTDB - Genome Taxonomy Database - Release R07-RS207" |
| file |  |
| 0 | "<https://data.ace.uq.edu.au/public/gtdb/data/releases/release207/207.0/genomic_files_reps/bac120_ssu_reps_r207.tar.gz>" |
| 1 | "<https://data.ace.uq.edu.au/public/gtdb/data/releases/release207/207.0/genomic_files_reps/ar53_ssu_reps_r207.tar.gz>" |
| citation | "Parks DH, Chuvochina M, Waite DW, Rinke C, Skarshewski A, Chaumeil PA, Hugenholtz P. A standardized bacterial taxonomy based on genome phylogeny substantially revises the tree of life. Nat Biotechnol. 2018 Nov;36(10):996-1004. doi: 10.1038/nbt.4229. Epub 2018 Aug 27. PMID: 30148503." |
| fmtscript | "taxref_reformat_gtdb.sh" |
| dbversion | "GTDB R07-RS207 (<https://data.ace.uq.edu.au/public/gtdb/data/releases/release207/207.0>)" |
| gtdb=R06-RS202 |  |
| title | "GTDB - Genome Taxonomy Database - Release R06-RS202" |
| file |  |
| 0 | "<https://data.ace.uq.edu.au/public/gtdb/data/releases/release202/202.0/genomic_files_reps/bac120_ssu_reps_r202.tar.gz>" |
| 1 | "<https://data.ace.uq.edu.au/public/gtdb/data/releases/release202/202.0/genomic_files_reps/ar122_ssu_reps_r202.tar.gz>" |
| citation | "Parks DH, Chuvochina M, Waite DW, Rinke C, Skarshewski A, Chaumeil PA, Hugenholtz P. A standardized bacterial taxonomy based on genome phylogeny substantially revises the tree of life. Nat Biotechnol. 2018 Nov;36(10):996-1004. doi: 10.1038/nbt.4229. Epub 2018 Aug 27. PMID: 30148503." |
| fmtscript | "taxref_reformat_gtdb.sh" |
| dbversion | "GTDB R06-RS202 (<https://data.ace.uq.edu.au/public/gtdb/data/releases/release202/202.0/>)" |
| gtdb=R05-RS95 |  |
| title | "GTDB - Genome Taxonomy Database - Release R05-RS95" |
| file |  |
| 0 | "<https://data.ace.uq.edu.au/public/gtdb/data/releases/release95/95.0/genomic_files_reps/bac120_ssu_reps_r95.tar.gz>" |
| 1 | "<https://data.ace.uq.edu.au/public/gtdb/data/releases/release95/95.0/genomic_files_reps/ar122_ssu_reps_r95.tar.gz>" |
| citation | "Parks DH, Chuvochina M, Waite DW, Rinke C, Skarshewski A, Chaumeil PA, Hugenholtz P. A standardized bacterial taxonomy based on genome phylogeny substantially revises the tree of life. Nat Biotechnol. 2018 Nov;36(10):996-1004. doi: 10.1038/nbt.4229. Epub 2018 Aug 27. PMID: 30148503." |
| fmtscript | "taxref_reformat_gtdb.sh" |
| dbversion | "GTDB R05-RS95 (<https://data.ace.uq.edu.au/public/gtdb/data/releases/release95/95.0/>)" |
| midori2-co1 |  |
| title | "MIDORI2 - CO1 Taxonomy Database - Release GB250" |
| file |  |
| 0 | "<http://reference-midori.info/download/Databases/GenBank250/DADA2_sp/uniq/MIDORI2_UNIQ_NUC_SP_GB250_CO1_DADA2.fasta.gz>" |
| citation | "Machida RJ, Leray M, Ho SL, Knowlton N. Metazoan mitochondrial gene sequence reference datasets for taxonomic assignment of environmental samples. Sci Data. 2017 Mar 14;4:170027. doi: 10.1038/sdata.2017.27. PMID: 28291235; PMCID: PMC5349245." |
| fmtscript | "taxref_reformat_midori2.sh" |
| dbversion | "MIDORI2-CO1 GB250 (<http://reference-midori.info/download/Databases/GenBank250/DADA2_sp/uniq/MIDORI2_UNIQ_NUC_SP_GB250_CO1_DADA2.fasta.gz>)" |
| taxlevels | "Phylum,Class,Order,Family,Genus,Species" |
| midori2-co1=gb250 |  |
| title | "MIDORI2 - CO1 Taxonomy Database - Release GB250" |
| file |  |
| 0 | "<http://reference-midori.info/download/Databases/GenBank250/DADA2_sp/uniq/MIDORI2_UNIQ_NUC_SP_GB250_CO1_DADA2.fasta.gz>" |
| citation | "Machida RJ, Leray M, Ho SL, Knowlton N. Metazoan mitochondrial gene sequence reference datasets for taxonomic assignment of environmental samples. Sci Data. 2017 Mar 14;4:170027. doi: 10.1038/sdata.2017.27. PMID: 28291235; PMCID: PMC5349245." |
| fmtscript | "taxref_reformat_midori2.sh" |
| dbversion | "MIDORI2-CO1 GB250 (<http://reference-midori.info/download/Databases/GenBank250/DADA2_sp/uniq/MIDORI2_UNIQ_NUC_SP_GB250_CO1_DADA2.fasta.gz>)" |
| taxlevels | "Phylum,Class,Order,Family,Genus,Species" |
| phytoref |  |
| title | "PhytoRef plastid 16S rRNA database for photosynthetic eukaryotes" |
| file |  |
| 0 | "<http://phytoref.sb-roscoff.fr/static/downloads/PhytoRef_with_taxonomy.fasta>" |
| citation | "Decelle, Johan, Sarah Romac, Rowena F. Stern, El Mahdi Bendif, Adriana Zingone, Stéphane Audic, Michael D. Guiry, et al. 2015. PhytoREF: A Reference Database of the Plastidial 16S rRNA Gene of Photosynthetic Eukaryotes with Curated Taxonomy. Molecular Ecology Resources 15 (6): 1435–45. <https://doi.org/10.1111/1755-0998.12401>." |
| fmtscript | "taxref_reformat_phytoref.sh" |
| dbversion | "unversioned" |
| taxlevels | "Domain,Supergroup,Subphylum,Class,Subclass,Order,Suborder,Family,Genus,Species" |
| pr2 |  |
| title | "PR2 - Protist Reference Ribosomal Database - Version 5.0.0" |
| file |  |
| 0 | "<https://github.com/pr2database/pr2database/releases/download/v5.0.0/pr2_version_5.0.0_SSU_dada2.fasta.gz>" |
| 1 | "<https://github.com/pr2database/pr2database/releases/download/v5.0.0/pr2_version_5.0.0_SSU_UTAX.fasta.gz>" |
| citation | "Guillou L, Bachar D, Audic S, Bass D, Berney C, Bittner L, Boutte C, Burgaud G, de Vargas C, Decelle J, Del Campo J, Dolan JR, Dunthorn M, Edvardsen B, Holzmann M, Kooistra WH, Lara E, Le Bescot N, Logares R, Mahé F, Massana R, Montresor M, Morard R, Not F, Pawlowski J, Probert I, Sauvadet AL, Siano R, Stoeck T, Vaulot D, Zimmermann P, Christen R. The Protist Ribosomal Reference database (PR2): a catalog of unicellular eukaryote small sub-unit rRNA sequences with curated taxonomy. Nucleic Acids Res. 2013 Jan;41(Database issue):D597-604. doi: 10.1093/nar/gks1160. Epub 2012 Nov 27. PMID: 23193267; PMCID: PMC3531120." |
| fmtscript | "taxref_reformat_pr2.sh" |
| dbversion | "PR2 v5.0.0 (<https://github.com/pr2database/pr2database/releases/tag/v5.0.0>)" |
| taxlevels | "Domain,Supergroup,Division,Subdivision,Class,Order,Family,Genus,Species" |
| pr2=5.0.0 |  |
| title | "PR2 - Protist Reference Ribosomal Database - Version 5.0.0" |
| file |  |
| 0 | "<https://github.com/pr2database/pr2database/releases/download/v5.0.0/pr2_version_5.0.0_SSU_dada2.fasta.gz>" |
| 1 | "<https://github.com/pr2database/pr2database/releases/download/v5.0.0/pr2_version_5.0.0_SSU_UTAX.fasta.gz>" |
| citation | "Guillou L, Bachar D, Audic S, Bass D, Berney C, Bittner L, Boutte C, Burgaud G, de Vargas C, Decelle J, Del Campo J, Dolan JR, Dunthorn M, Edvardsen B, Holzmann M, Kooistra WH, Lara E, Le Bescot N, Logares R, Mahé F, Massana R, Montresor M, Morard R, Not F, Pawlowski J, Probert I, Sauvadet AL, Siano R, Stoeck T, Vaulot D, Zimmermann P, Christen R. The Protist Ribosomal Reference database (PR2): a catalog of unicellular eukaryote small sub-unit rRNA sequences with curated taxonomy. Nucleic Acids Res. 2013 Jan;41(Database issue):D597-604. doi: 10.1093/nar/gks1160. Epub 2012 Nov 27. PMID: 23193267; PMCID: PMC3531120." |
| fmtscript | "taxref_reformat_pr2.sh" |
| dbversion | "PR2 v5.0.0 (<https://github.com/pr2database/pr2database/releases/tag/v5.0.0>)" |
| taxlevels | "Domain,Supergroup,Division,Subdivision,Class,Order,Family,Genus,Species" |
| pr2=4.14.0 |  |
| title | "PR2 - Protist Reference Ribosomal Database - Version 4.14.0" |
| file |  |
| 0 | "<https://github.com/pr2database/pr2database/releases/download/v4.14.0/pr2_version_4.14.0_SSU_dada2.fasta.gz>" |
| 1 | "<https://github.com/pr2database/pr2database/releases/download/v4.14.0/pr2_version_4.14.0_SSU_UTAX.fasta.gz>" |
| citation | "Guillou L, Bachar D, Audic S, Bass D, Berney C, Bittner L, Boutte C, Burgaud G, de Vargas C, Decelle J, Del Campo J, Dolan JR, Dunthorn M, Edvardsen B, Holzmann M, Kooistra WH, Lara E, Le Bescot N, Logares R, Mahé F, Massana R, Montresor M, Morard R, Not F, Pawlowski J, Probert I, Sauvadet AL, Siano R, Stoeck T, Vaulot D, Zimmermann P, Christen R. The Protist Ribosomal Reference database (PR2): a catalog of unicellular eukaryote small sub-unit rRNA sequences with curated taxonomy. Nucleic Acids Res. 2013 Jan;41(Database issue):D597-604. doi: 10.1093/nar/gks1160. Epub 2012 Nov 27. PMID: 23193267; PMCID: PMC3531120." |
| fmtscript | "taxref_reformat_pr2.sh" |
| dbversion | "PR2 v4.14.0 (<https://github.com/pr2database/pr2database/releases/tag/v4.14.0>)" |
| taxlevels | "Domain,Kingdom,Phylum,Class,Order,Family,Genus,Species" |
| pr2=4.13.0 |  |
| title | "PR2 - Protist Reference Ribosomal Database - Version 4.13.0" |
| file |  |
| 0 | "<https://github.com/pr2database/pr2database/releases/download/v4.13.0/pr2_version_4.13.0_18S_dada2.fasta.gz>" |
| 1 | "<https://github.com/pr2database/pr2database/releases/download/v4.13.0/pr2_version_4.13.0_18S_UTAX.fasta.gz>" |
| citation | "Guillou L, Bachar D, Audic S, Bass D, Berney C, Bittner L, Boutte C, Burgaud G, de Vargas C, Decelle J, Del Campo J, Dolan JR, Dunthorn M, Edvardsen B, Holzmann M, Kooistra WH, Lara E, Le Bescot N, Logares R, Mahé F, Massana R, Montresor M, Morard R, Not F, Pawlowski J, Probert I, Sauvadet AL, Siano R, Stoeck T, Vaulot D, Zimmermann P, Christen R. The Protist Ribosomal Reference database (PR2): a catalog of unicellular eukaryote small sub-unit rRNA sequences with curated taxonomy. Nucleic Acids Res. 2013 Jan;41(Database issue):D597-604. doi: 10.1093/nar/gks1160. Epub 2012 Nov 27. PMID: 23193267; PMCID: PMC3531120." |
| fmtscript | "taxref_reformat_pr2.sh" |
| dbversion | "PR2 v4.13.0 (<https://github.com/pr2database/pr2database/releases/tag/v4.13.0>)" |
| taxlevels | "Domain,Kingdom,Phylum,Class,Order,Family,Genus,Species" |
| rdp |  |
| title | "RDP - Ribosomal Database Project - RDP trainset 18/release 11.5" |
| file |  |
| 0 | "<https://zenodo.org/record/4310151/files/rdp_train_set_18.fa.gz>" |
| 1 | "<https://zenodo.org/record/4310151/files/rdp_species_assignment_18.fa.gz>" |
| citation | "Cole JR, Wang Q, Fish JA, Chai B, McGarrell DM, Sun Y, Brown CT, Porras-Alfaro A, Kuske CR, Tiedje JM. Ribosomal Database Project: data and tools for high throughput rRNA analysis. Nucleic Acids Res. 2014 Jan;42(Database issue):D633-42. doi: 10.1093/nar/gkt1244. Epub 2013 Nov 27. PMID: 24288368; PMCID: PMC3965039." |
| fmtscript | "taxref_reformat_standard.sh" |
| dbversion | "RDP 18/11.5 (<https://zenodo.org/record/4310151/>)" |
| rdp=18 |  |
| title | "RDP - Ribosomal Database Project - RDP trainset 18/release 11.5" |
| file |  |
| 0 | "<https://zenodo.org/record/4310151/files/rdp_train_set_18.fa.gz>" |
| 1 | "<https://zenodo.org/record/4310151/files/rdp_species_assignment_18.fa.gz>" |
| citation | "Cole JR, Wang Q, Fish JA, Chai B, McGarrell DM, Sun Y, Brown CT, Porras-Alfaro A, Kuske CR, Tiedje JM. Ribosomal Database Project: data and tools for high throughput rRNA analysis. Nucleic Acids Res. 2014 Jan;42(Database issue):D633-42. doi: 10.1093/nar/gkt1244. Epub 2013 Nov 27. PMID: 24288368; PMCID: PMC3965039." |
| fmtscript | "taxref_reformat_standard.sh" |
| dbversion | "RDP 18/11.5 (<https://zenodo.org/record/4310151/>)" |
| sbdi-gtdb |  |
| title | "SBDI-GTDB - Sativa curated 16S GTDB database - Release R07-RS207-1" |
| file |  |
| 0 | "<https://scilifelab.figshare.com/ndownloader/files/36980767>" |
| 1 | "<https://scilifelab.figshare.com/ndownloader/files/36980788>" |
| citation | "Lundin D, Andersson A. SBDI Sativa curated 16S GTDB database. FigShare. doi: 10.17044/scilifelab.14869077.v4" |
| fmtscript | "taxref_reformat_sbdi-gtdb.sh" |
| dbversion | "SBDI-GTDB-R07-RS207-1 (<https://scilifelab.figshare.com/articles/dataset/SBDI_Sativa_curated_16S_GTDB_database/14869077/4>)" |
| taxlevels | "Domain,Kingdom,Phylum,Class,Order,Family,Genus,Species" |
| sbdi-gtdb=R07-RS207-1 |  |
| title | "SBDI-GTDB - Sativa curated 16S GTDB database - Release R07-RS207-1" |
| file |  |
| 0 | "<https://scilifelab.figshare.com/ndownloader/files/36980767>" |
| 1 | "<https://scilifelab.figshare.com/ndownloader/files/36980788>" |
| citation | "Lundin D, Andersson A. SBDI Sativa curated 16S GTDB database. FigShare. doi: 10.17044/scilifelab.14869077.v4" |
| fmtscript | "taxref_reformat_sbdi-gtdb.sh" |
| dbversion | "SBDI-GTDB-R07-RS207-1 (<https://scilifelab.figshare.com/articles/dataset/SBDI_Sativa_curated_16S_GTDB_database/14869077/4>)" |
| taxlevels | "Domain,Kingdom,Phylum,Class,Order,Family,Genus,Species" |
| sbdi-gtdb=R06-RS202-3 |  |
| title | "SBDI-GTDB - Sativa curated 16S GTDB database - Release R06-RS202-1" |
| file |  |
| 0 | "<https://scilifelab.figshare.com/ndownloader/files/31370437>" |
| 1 | "<https://scilifelab.figshare.com/ndownloader/files/31370434>" |
| citation | "Lundin D, Andersson A. SBDI Sativa curated 16S GTDB database. FigShare. doi: 10.17044/scilifelab.14869077.v3" |
| fmtscript | "taxref_reformat_sbdi-gtdb.sh" |
| dbversion | "SBDI-GTDB-R06-RS202-3 (<https://scilifelab.figshare.com/articles/dataset/SBDI_Sativa_curated_16S_GTDB_database/14869077/3>)" |
| taxlevels | "Domain,Kingdom,Phylum,Class,Order,Family,Genus,Species" |
| sbdi-gtdb=R06-RS202-1 |  |
| title | "SBDI-GTDB - Sativa curated 16S GTDB database - Release R06-RS202-1" |
| file |  |
| 0 | "<https://scilifelab.figshare.com/ndownloader/files/28624479>" |
| 1 | "<https://scilifelab.figshare.com/ndownloader/files/28624482>" |
| citation | "Lundin D, Andersson A. SBDI Sativa curated 16S GTDB database. FigShare. doi: 10.17044/scilifelab.14869077.v1" |
| fmtscript | "taxref_reformat_sbdi-gtdb.sh" |
| dbversion | "SBDI-GTDB-R06-RS202-1 (<https://scilifelab.figshare.com/articles/dataset/SBDI_Sativa_curated_16S_GTDB_database/14869077/1>)" |
| taxlevels | "Domain,Kingdom,Phylum,Class,Order,Family,Genus,Species" |
| silva |  |
| title | "Silva 138.1 prokaryotic SSU" |
| file |  |
| 0 | "<https://zenodo.org/record/4587955/files/silva_nr99_v138.1_wSpecies_train_set.fa.gz>" |
| 1 | "<https://zenodo.org/record/4587955/files/silva_species_assignment_v138.1.fa.gz>" |
| citation | "Quast C, Pruesse E, Yilmaz P, Gerken J, Schweer T, Yarza P, Peplies J, Glöckner FO. The SILVA ribosomal RNA gene database project: improved data processing and web-based tools. Nucleic Acids Res. 2013 Jan;41(Database issue):D590-6. doi: 10.1093/nar/gks1219. Epub 2012 Nov 28. PMID: 23193283; PMCID: PMC3531112." |
| fmtscript | "taxref_reformat_standard.sh" |
| dbversion | "SILVA v138.1 (<https://zenodo.org/record/4587955>)" |
| silva=138 |  |
| title | "Silva 138.1 prokaryotic SSU" |
| file |  |
| 0 | "<https://zenodo.org/record/4587955/files/silva_nr99_v138.1_wSpecies_train_set.fa.gz>" |
| 1 | "<https://zenodo.org/record/4587955/files/silva_species_assignment_v138.1.fa.gz>" |
| citation | "Quast C, Pruesse E, Yilmaz P, Gerken J, Schweer T, Yarza P, Peplies J, Glöckner FO. The SILVA ribosomal RNA gene database project: improved data processing and web-based tools. Nucleic Acids Res. 2013 Jan;41(Database issue):D590-6. doi: 10.1093/nar/gks1219. Epub 2012 Nov 28. PMID: 23193283; PMCID: PMC3531112." |
| fmtscript | "taxref_reformat_standard.sh" |
| dbversion | "SILVA v138.1 (<https://zenodo.org/record/4587955>)" |
| silva=132 |  |
| title | "Silva Project's version 132 release" |
| file |  |
| 0 | "<https://zenodo.org/record/1172783/files/silva_nr_v132_train_set.fa.gz>" |
| 1 | "<https://zenodo.org/record/1172783/files/silva_species_assignment_v132.fa.gz>" |
| citation | "Quast C, Pruesse E, Yilmaz P, Gerken J, Schweer T, Yarza P, Peplies J, Glöckner FO. The SILVA ribosomal RNA gene database project: improved data processing and web-based tools. Nucleic Acids Res. 2013 Jan;41(Database issue):D590-6. doi: 10.1093/nar/gks1219. Epub 2012 Nov 28. PMID: 23193283; PMCID: PMC3531112." |
| fmtscript | "taxref_reformat_standard.sh" |
| dbversion | "SILVA v132 (<https://zenodo.org/record/1172783>)" |
| unite-fungi |  |
| title | "UNITE general FASTA release for Fungi - Version 9.0" |
| file |  |
| 0 | "<https://files.plutof.ut.ee/public/orig/1E/25/1E25CA4CC30A31C2E2B8CB2C89824C83D080A7F5A62E6263A0E95B37C6628067.tgz>" |
| citation | "Abarenkov, Kessy; Zirk, Allan; Piirmann, Timo; Pöhönen, Raivo; Ivanov, Filipp; Nilsson, R. Henrik; Kõljalg, Urmas (2022): UNITE general FASTA release for Fungi. Version 16.10.2022. UNITE Community. <https://doi.org/10.15156/BIO/2483911>" |
| fmtscript | "taxref_reformat_unite.sh" |
| dbversion | "UNITE-fungi v9.0 (<https://doi.org/10.15156/BIO/2483911>)" |
| shfile |  |
| 0 | "<https://figshare.scilifelab.se/ndownloader/files/40788767>" |
| 1 | "<https://figshare.scilifelab.se/ndownloader/files/40788770>" |
| unite-fungi=9.0 |  |
| title | "UNITE general FASTA release for Fungi - Version 9.0" |
| file |  |
| 0 | "<https://files.plutof.ut.ee/public/orig/1E/25/1E25CA4CC30A31C2E2B8CB2C89824C83D080A7F5A62E6263A0E95B37C6628067.tgz>" |
| citation | "Abarenkov, Kessy; Zirk, Allan; Piirmann, Timo; Pöhönen, Raivo; Ivanov, Filipp; Nilsson, R. Henrik; Kõljalg, Urmas (2022): UNITE general FASTA release for Fungi. Version 16.10.2022. UNITE Community. <https://doi.org/10.15156/BIO/2483911>" |
| fmtscript | "taxref_reformat_unite.sh" |
| dbversion | "UNITE-fungi v9.0 (<https://doi.org/10.15156/BIO/2483911>)" |
| shfile |  |
| 0 | "<https://figshare.scilifelab.se/ndownloader/files/40788767>" |
| 1 | "<https://figshare.scilifelab.se/ndownloader/files/40788770>" |
| unite-fungi=8.3 |  |
| title | "UNITE general FASTA release for Fungi - Version 8.3" |
| file |  |
| 0 | "<https://files.plutof.ut.ee/public/orig/7B/23/7B235835FAF5C85D7B01E40FEF17F687914CB81A182554C5BD95E3168328E604.tgz>" |
| citation | "Abarenkov, Kessy; Zirk, Allan; Piirmann, Timo; Pöhönen, Raivo; Ivanov, Filipp; Nilsson, R. Henrik; Kõljalg, Urmas (2021): UNITE general FASTA release for Fungi. UNITE Community. 10.15156/BIO/1280049" |
| fmtscript | "taxref_reformat_unite.sh" |
| dbversion | "UNITE-fungi v8.3 (<https://doi.org/10.15156/BIO/1280049>)" |
| shfile |  |
| 0 | "<https://scilifelab.figshare.com/ndownloader/files/34497977>" |
| 1 | "<https://scilifelab.figshare.com/ndownloader/files/34497980>" |
| unite-fungi=8.2 |  |
| title | "UNITE general FASTA release for Fungi - Version 8.2" |
| file |  |
| 0 | "<https://files.plutof.ut.ee/public/orig/E7/28/E728E2CAB797C90A01CD271118F574B8B7D0DAEAB7E81193EB89A2AC769A0896.gz>" |
| citation | "Abarenkov, Kessy; Zirk, Allan; Piirmann, Timo; Pöhönen, Raivo; Ivanov, Filipp; Nilsson, R. Henrik; Kõljalg, Urmas (2020): UNITE general FASTA release for Fungi. UNITE Community. 10.15156/BIO/786368" |
| fmtscript | "taxref_reformat_unite.sh" |
| dbversion | "UNITE-fungi v8.2 (<https://doi.org/10.15156/BIO/786368>)" |
| shfile |  |
| 0 | "<https://scilifelab.figshare.com/ndownloader/files/34497971>" |
| 1 | "<https://scilifelab.figshare.com/ndownloader/files/34497974>" |
| unite-alleuk |  |
| title | "UNITE general FASTA release for eukaryotes - Version 9.0" |
| file |  |
| 0 | "<https://files.plutof.ut.ee/public/orig/F2/62/F262D942DEB8CAB3AEB9F313F67B04050E364B72E6707F99755DDCB271C45A48.tgz>" |
| citation | "Abarenkov, Kessy; Zirk, Allan; Piirmann, Timo; Pöhönen, Raivo; Ivanov, Filipp; Nilsson, R. Henrik; Kõljalg, Urmas (2022): UNITE general FASTA release for eukaryotes. Version 16.10.2022. UNITE Community. <https://doi.org/10.15156/BIO/2483913>" |
| fmtscript | "taxref_reformat_unite.sh" |
| dbversion | "UNITE-alleuk v9.0 (<https://doi.org/10.15156/BIO/2483913>)" |
| shfile |  |
| 0 | "<https://figshare.scilifelab.se/ndownloader/files/40788773>" |
| 1 | "<https://figshare.scilifelab.se/ndownloader/files/40788776>" |
| unite-alleuk=9.0 |  |
| title | "UNITE general FASTA release for eukaryotes - Version 9.0" |
| file |  |
| 0 | "<https://files.plutof.ut.ee/public/orig/F2/62/F262D942DEB8CAB3AEB9F313F67B04050E364B72E6707F99755DDCB271C45A48.tgz>" |
| citation | "Abarenkov, Kessy; Zirk, Allan; Piirmann, Timo; Pöhönen, Raivo; Ivanov, Filipp; Nilsson, R. Henrik; Kõljalg, Urmas (2022): UNITE general FASTA release for eukaryotes. Version 16.10.2022. UNITE Community. <https://doi.org/10.15156/BIO/2483913>" |
| fmtscript | "taxref_reformat_unite.sh" |
| dbversion | "UNITE-alleuk v9.0 (<https://doi.org/10.15156/BIO/2483913>)" |
| shfile |  |
| 0 | "<https://figshare.scilifelab.se/ndownloader/files/40788773>" |
| 1 | "<https://figshare.scilifelab.se/ndownloader/files/40788776>" |
| unite-alleuk=8.3 |  |
| title | "UNITE general FASTA release for eukaryotes - Version 8.3" |
| file |  |
| 0 | "<https://files.plutof.ut.ee/public/orig/E5/F5/E5F5E426DEC78BA2F7EC530621DDBD3F10564A09CBC2A5C4D3B3CBE7E37C5E1A.tgz>" |
| citation | "Abarenkov, Kessy; Zirk, Allan; Piirmann, Timo; Pöhönen, Raivo; Ivanov, Filipp; Nilsson, R. Henrik; Kõljalg, Urmas (2021): UNITE general FASTA release for eukaryotes. UNITE Community. 10.15156/BIO/1280127" |
| fmtscript | "taxref_reformat_unite.sh" |
| dbversion | "UNITE-alleuk v8.3 (<https://doi.org/10.15156/BIO/1280127>)" |
| shfile |  |
| 0 | "<https://scilifelab.figshare.com/ndownloader/files/34994575>" |
| 1 | "<https://scilifelab.figshare.com/ndownloader/files/34994578>" |
| unite-alleuk=8.2 |  |
| title | "UNITE general FASTA release for eukaryotes - Version 8.2" |
| file |  |
| 0 | "<https://files.plutof.ut.ee/public/orig/F9/ED/F9EDE36E5209F469056675EBD672425BC06EACB7FE0C0D18F5A13E4CA632DCFA.gz>" |
| citation | "Abarenkov, Kessy; Zirk, Allan; Piirmann, Timo; Pöhönen, Raivo; Ivanov, Filipp; Nilsson, R. Henrik; Kõljalg, Urmas (2020): UNITE general FASTA release for eukaryotes. UNITE Community. 10.15156/BIO/786370" |
| fmtscript | "taxref_reformat_unite.sh" |
| dbversion | "UNITE-alleuk v8.2 (<https://doi.org/10.15156/BIO/786370>)" |
| shfile |  |
| 0 | "<https://scilifelab.figshare.com/ndownloader/files/34994569>" |
| 1 | "<https://scilifelab.figshare.com/ndownloader/files/34994572>" |
| zehr-nifh |  |
| title | "Zehr lab nifH database - version 2.5.0" |
| file |  |
| 0 | "<https://raw.githubusercontent.com/moyn413/nifHdada2/master/nifH_dada2_v2.0.5.fasta>" |
| 1 | "<https://raw.githubusercontent.com/moyn413/nifHdada2/master/nifH_dada2_phylum_v2.0.5.csv>" |
| citation | "M. A. Moynihan & C. Furbo Reeder 2023. nifHdada2 GitHub repository, v2.0.5. Zenodo. <http://doi.org/10.5281/zenodo.7996213>" |
| fmtscript | "taxref_reformat_zehr-nifh.sh" |
| dbversion | "Zehr-nifH v. 2.5.0" |
| taxlevels | "Domain,Phylum,Class,Order,Family,Genus" |
| zehr-nifh=2.5.0 |  |
| title | "Zehr lab nifH database - version 2.5.0" |
| file |  |
| 0 | "<https://raw.githubusercontent.com/moyn413/nifHdada2/master/nifH_dada2_v2.0.5.fasta>" |
| 1 | "<https://raw.githubusercontent.com/moyn413/nifHdada2/master/nifH_dada2_phylum_v2.0.5.csv>" |
| citation | "M. A. Moynihan & C. Furbo Reeder 2023. nifHdada2 GitHub repository, v2.0.5. Zenodo. <http://doi.org/10.5281/zenodo.7996213>" |
| fmtscript | "taxref_reformat_zehr-nifh.sh" |
| dbversion | "Zehr-nifH v. 2.5.0" |
| taxlevels | "Domain,Phylum,Class,Order,Family,Genus" |
| qiime_ref_databases |  |
| silva=138 |  |
| title | "QIIME2 pre-formatted SILVA dereplicated at 99% similarity - Version 138" |
| file |  |
| 0 | "<https://data.qiime2.org/2023.7/common/silva-138-99-seqs.qza>" |
| 1 | "<https://data.qiime2.org/2023.7/common/silva-138-99-tax.qza>" |
| citation | "<https://www.arb-silva.de/>; Bokulich, N.A., Robeson, M., Dillon, M.R. bokulich-lab/RESCRIPt. Zenodo. <http://doi.org/10.5281/zenodo.3891931>" |
| license | "<https://www.arb-silva.de/silva-license-information/>" |
| fmtscript | "taxref_reformat_qiime_silva138.sh" |
| silva |  |
| title | "QIIME2 pre-formatted SILVA dereplicated at 99% similarity - Version 138" |
| file |  |
| 0 | "<https://data.qiime2.org/2023.7/common/silva-138-99-seqs.qza>" |
| 1 | "<https://data.qiime2.org/2023.7/common/silva-138-99-tax.qza>" |
| citation | "<https://www.arb-silva.de/>; Bokulich, N.A., Robeson, M., Dillon, M.R. bokulich-lab/RESCRIPt. Zenodo. <http://doi.org/10.5281/zenodo.3891931>" |
| license | "<https://www.arb-silva.de/silva-license-information/>" |
| fmtscript | "taxref_reformat_qiime_silva138.sh" |
| unite-fungi |  |
| title | "UNITE QIIME release for Fungi - Version 8.3" |
| file |  |
| 0 | "<https://files.plutof.ut.ee/public/orig/C5/54/C5547B97AAA979E45F79DC4C8C4B12113389343D7588716B5AD330F8BDB300C9.tgz>" |
| citation | "Abarenkov, Kessy; Zirk, Allan; Piirmann, Timo; Pöhönen, Raivo; Ivanov, Filipp; Nilsson, R. Henrik; Kõljalg, Urmas (2021): UNITE QIIME release for Fungi. Version 10.05.2021. UNITE Community. <https://doi.org/10.15156/BIO/1264708>" |
| fmtscript | "taxref_reformat_qiime_unite.sh" |
| unite-fungi=8.3 |  |
| title | "UNITE QIIME release for Fungi - Version 8.3" |
| file |  |
| 0 | "<https://files.plutof.ut.ee/public/orig/C5/54/C5547B97AAA979E45F79DC4C8C4B12113389343D7588716B5AD330F8BDB300C9.tgz>" |
| citation | "Abarenkov, Kessy; Zirk, Allan; Piirmann, Timo; Pöhönen, Raivo; Ivanov, Filipp; Nilsson, R. Henrik; Kõljalg, Urmas (2021): UNITE QIIME release for Fungi. Version 10.05.2021. UNITE Community. <https://doi.org/10.15156/BIO/1264708>" |
| fmtscript | "taxref_reformat_qiime_unite.sh" |
| unite-fungi=8.2 |  |
| title | "UNITE QIIME release for Fungi - Version 8.2" |
| file |  |
| 0 | "<https://files.plutof.ut.ee/public/orig/98/AE/98AE96C6593FC9C52D1C46B96C2D9064291F4DBA625EF189FEC1CCAFCF4A1691.gz>" |
| citation | "Abarenkov, Kessy; Zirk, Allan; Piirmann, Timo; Pöhönen, Raivo; Ivanov, Filipp; Nilsson, R. Henrik; Kõljalg, Urmas (2020): UNITE QIIME release for Fungi. Version 04.02.2020. UNITE Community. <https://doi.org/10.15156/BIO/786385>" |
| fmtscript | "taxref_reformat_qiime_unite.sh" |
| unite-alleuk |  |
| title | "UNITE QIIME release for eukaryotes - Version 9.0" |
| file |  |
| 0 | "<https://files.plutof.ut.ee/public/orig/8F/FC/8FFCC8A730E50FEEF8CFFEEFEF02A22FBCF7E02B7FD31C6649754834D2CB0E6F.tgz>" |
| citation | "Abarenkov, Kessy; Zirk, Allan; Piirmann, Timo; Pöhönen, Raivo; Ivanov, Filipp; Nilsson, R. Henrik; Kõljalg, Urmas (2022): UNITE QIIME release for eukaryotes. Version 16.10.2022. UNITE Community. <https://doi.org/10.15156/BIO/2483917>" |
| fmtscript | "taxref_reformat_qiime_unite.sh" |
| unite-alleuk=9.0 |  |
| title | "UNITE QIIME release for eukaryotes - Version 9.0" |
| file |  |
| 0 | "<https://files.plutof.ut.ee/public/orig/8F/FC/8FFCC8A730E50FEEF8CFFEEFEF02A22FBCF7E02B7FD31C6649754834D2CB0E6F.tgz>" |
| citation | "Abarenkov, Kessy; Zirk, Allan; Piirmann, Timo; Pöhönen, Raivo; Ivanov, Filipp; Nilsson, R. Henrik; Kõljalg, Urmas (2022): UNITE QIIME release for eukaryotes. Version 16.10.2022. UNITE Community. <https://doi.org/10.15156/BIO/2483917>" |
| fmtscript | "taxref_reformat_qiime_unite.sh" |
| unite-alleuk=8.3 |  |
| title | "UNITE QIIME release for eukaryotes - Version 8.3" |
| file |  |
| 0 | "<https://files.plutof.ut.ee/public/orig/48/29/4829D91F763E20F0F4376A60AA53FC9FBE6029A7D1BDC1B45347DD64EDE5D560.tgz>" |
| citation | "Abarenkov, Kessy; Zirk, Allan; Piirmann, Timo; Pöhönen, Raivo; Ivanov, Filipp; Nilsson, R. Henrik; Kõljalg, Urmas (2021): UNITE QIIME release for eukaryotes. Version 10.05.2021. UNITE Community. <https://doi.org/10.15156/BIO/1264819>" |
| fmtscript | "taxref_reformat_qiime_unite.sh" |
| unite-alleuk=8.2 |  |
| title | "UNITE QIIME release for eukaryotes - Version 8.2" |
| file |  |
| 0 | "<https://files.plutof.ut.ee/public/orig/6E/0E/6E0EDD5592003B47C70A1B384C3C784AA32B726AC861CD7E2BD22AEB0278675E.gz>" |
| citation | "Abarenkov, Kessy; Zirk, Allan; Piirmann, Timo; Pöhönen, Raivo; Ivanov, Filipp; Nilsson, R. Henrik; Kõljalg, Urmas (2020): UNITE QIIME release for eukaryotes. Version 04.02.2020. UNITE Community. <https://doi.org/10.15156/BIO/786386>" |
| fmtscript | "taxref_reformat_qiime_unite.sh" |
| greengenes85 |  |
| title | "Greengenes 16S - Version 13_8 - clustered at 85% similarity - for testing purposes only" |
| file |  |
| 0 | "<https://data.qiime2.org/2023.7/tutorials/training-feature-classifiers/85_otus.fasta>" |
| 1 | "<https://data.qiime2.org/2023.7/tutorials/training-feature-classifiers/85_otu_taxonomy.txt>" |
| citation | "McDonald, D., Price, M., Goodrich, J. et al. An improved Greengenes taxonomy with explicit ranks for ecological and evolutionary analyses of bacteria and archaea. ISME J 6, 610–618 (2012). <https://doi.org/10.1038/ismej.2011.139>" |
| fmtscript | "taxref_reformat_qiime_greengenes85.sh" |
| greengenes2 |  |
| title | "Greengenes2 16S - Version 2022.10" |
| file |  |
| 0 | "<http://ftp.microbio.me/greengenes_release/2022.10/2022.10.seqs.fna.gz>" |
| 1 | "<http://ftp.microbio.me/greengenes_release/2022.10/2022.10.taxonomy.md5.tsv.gz>" |
| citation | "McDonald, D., Jiang, Y., Balaban, M. et al. Greengenes2 unifies microbial data in a single reference tree. Nat Biotechnol (2023). <https://doi.org/10.1038/s41587-023-01845-1>" |
| fmtscript | "taxref_reformat_qiime_greengenes2022.sh" |
| greengenes2=2022.10 |  |
| title | "Greengenes2 16S - Version 2022.10" |
| file |  |
| 0 | "<http://ftp.microbio.me/greengenes_release/2022.10/2022.10.seqs.fna.gz>" |
| 1 | "<http://ftp.microbio.me/greengenes_release/2022.10/2022.10.taxonomy.md5.tsv.gz>" |
| citation | "McDonald, D., Jiang, Y., Balaban, M. et al. Greengenes2 unifies microbial data in a single reference tree. Nat Biotechnol (2023). <https://doi.org/10.1038/s41587-023-01845-1>" |
| fmtscript | "taxref_reformat_qiime_greengenes2022.sh" |
| sintax_ref_databases |  |
| coidb |  |
| title | "COIDB - CO1 Taxonomy Database - Release 221216" |
| file |  |
| 0 | "<https://figshare.scilifelab.se/ndownloader/files/38787078>" |
| citation | "Sundh J, Manoharan L, Iwaszkiewicz-Eggebrecht E, Miraldo A, Andersson A, Ronquist F. COI reference sequences from BOLD DB. doi: <https://doi.org/10.17044/scilifelab.20514192.v2>" |
| fmtscript | "taxref_reformat_sintax.sh" |
| dbversion | "COIDB 221216 (<https://doi.org/10.17044/scilifelab.20514192.v2>)" |
| taxlevels | "Kingdom,Phylum,Class,Order,Family,Genus,Species,BOLD_bin" |
| coidb=221216 |  |
| title | "COIDB - CO1 Taxonomy Database - Release 221216" |
| file |  |
| 0 | "<https://figshare.scilifelab.se/ndownloader/files/38787078>" |
| citation | "Sundh J, Manoharan L, Iwaszkiewicz-Eggebrecht E, Miraldo A, Andersson A, Ronquist F. COI reference sequences from BOLD DB. doi: <https://doi.org/10.17044/scilifelab.20514192.v2>" |
| fmtscript | "taxref_reformat_sintax.sh" |
| dbversion | "COIDB 221216 (<https://doi.org/10.17044/scilifelab.20514192.v2>)" |
| taxlevels | "Kingdom,Phylum,Class,Order,Family,Genus,Species,BOLD_bin" |
| unite-fungi |  |
| title | "UNITE USEARCH/UTAX release for Fungi - Version 9.0" |
| file |  |
| 0 | "<https://files.plutof.ut.ee/public/orig/19/1B/191B0D889A6B7B05DF4C103B118ABB3E0CF8EDBEA5B3E3FAB3EAFE3B72D7F3C8.gz>" |
| citation | "Abarenkov, Kessy; Zirk, Allan; Piirmann, Timo; Pöhönen, Raivo; Ivanov, Filipp; Nilsson, R. Henrik; Kõljalg, Urmas (2022): UNITE USEARCH/UTAX release for Fungi. Version 16.10.2022. UNITE Community. <https://doi.org/10.15156/BIO/2483923>" |
| fmtscript | "taxref_reformat_sintax.sh" |
| dbversion | "UNITE-fungi v9.0 (<https://doi.org/10.15156/BIO/2483923>)" |
| unite-fungi=9.0 |  |
| title | "UNITE USEARCH/UTAX release for Fungi - Version 9.0" |
| file |  |
| 0 | "<https://files.plutof.ut.ee/public/orig/19/1B/191B0D889A6B7B05DF4C103B118ABB3E0CF8EDBEA5B3E3FAB3EAFE3B72D7F3C8.gz>" |
| citation | "Abarenkov, Kessy; Zirk, Allan; Piirmann, Timo; Pöhönen, Raivo; Ivanov, Filipp; Nilsson, R. Henrik; Kõljalg, Urmas (2022): UNITE USEARCH/UTAX release for Fungi. Version 16.10.2022. UNITE Community. <https://doi.org/10.15156/BIO/2483923>" |
| fmtscript | "taxref_reformat_sintax.sh" |
| dbversion | "UNITE-fungi v9.0 (<https://doi.org/10.15156/BIO/2483923>)" |
| unite-fungi=8.3 |  |
| title | "UNITE USEARCH/UTAX release for Fungi - Version 8.3" |
| file |  |
| 0 | "<https://files.plutof.ut.ee/public/orig/82/CB/82CB44BBAAA7D3AEAC297B5689BDA2963E8D0666E01FE0B54096147AFAF85263.gz>" |
| citation | "Abarenkov, Kessy; Zirk, Allan; Piirmann, Timo; Pöhönen, Raivo; Ivanov, Filipp; Nilsson, R. Henrik; Kõljalg, Urmas (2021): UNITE USEARCH/UTAX release for Fungi. UNITE Community. 10.15156/BIO/1280276" |
| fmtscript | "taxref_reformat_sintax.sh" |
| dbversion | "UNITE-fungi v8.3 (<https://dx.doi.org/10.15156/BIO/1280276>)" |
| unite-fungi=8.2 |  |
| title | "UNITE USEARCH/UTAX release for Fungi - Version 8.2" |
| file |  |
| 0 | "<https://files.plutof.ut.ee/public/orig/E8/83/E883EB19E3EA7B64C1F652521301239831FAFE0BFF015C9E2B4786DC0976C0FC.gz>" |
| citation | "Abarenkov, Kessy; Zirk, Allan; Piirmann, Timo; Pöhönen, Raivo; Ivanov, Filipp; Nilsson, R. Henrik; Kõljalg, Urmas (2020): UNITE USEARCH/UTAX release for Fungi. UNITE Community. 10.15156/BIO/786375" |
| fmtscript | "taxref_reformat_sintax.sh" |
| dbversion | "UNITE-fungi v8.2 (<https://doi.org/10.15156/BIO/786375>)" |
| unite-alleuk |  |
| title | "UNITE USEARCH/UTAX release for eukaryotes - Version 9.0" |
| file |  |
| 0 | "<https://files.plutof.ut.ee/public/orig/AB/8C/AB8C119FC82CF5AFAFCB93CA4FFFF2B42A03CF1275DE23F60B887392E8FDEA21.gz>" |
| citation | "Abarenkov, Kessy; Zirk, Allan; Piirmann, Timo; Pöhönen, Raivo; Ivanov, Filipp; Nilsson, R. Henrik; Kõljalg, Urmas (2022): UNITE USEARCH/UTAX release for eukaryotes. Version 16.10.2022. UNITE Community. <https://doi.org/10.15156/BIO/2483924>" |
| fmtscript | "taxref_reformat_sintax.sh" |
| dbversion | "UNITE-alleuk v9.0 (<https://doi.org/10.15156/BIO/2483924>)" |
| unite-alleuk=9.0 |  |
| title | "UNITE USEARCH/UTAX release for eukaryotes - Version 9.0" |
| file |  |
| 0 | "<https://files.plutof.ut.ee/public/orig/AB/8C/AB8C119FC82CF5AFAFCB93CA4FFFF2B42A03CF1275DE23F60B887392E8FDEA21.gz>" |
| citation | "Abarenkov, Kessy; Zirk, Allan; Piirmann, Timo; Pöhönen, Raivo; Ivanov, Filipp; Nilsson, R. Henrik; Kõljalg, Urmas (2022): UNITE USEARCH/UTAX release for eukaryotes. Version 16.10.2022. UNITE Community. <https://doi.org/10.15156/BIO/2483924>" |
| fmtscript | "taxref_reformat_sintax.sh" |
| dbversion | "UNITE-alleuk v9.0 (<https://doi.org/10.15156/BIO/2483924>)" |
| unite-alleuk=8.3 |  |
| title | "UNITE USEARCH/UTAX release for eukaryotes - Version 8.3" |
| file |  |
| 0 | "<https://files.plutof.ut.ee/public/orig/B9/35/B9351C91550A52713CB66DB7A1CEF35765310EBB23B6667AC93E714E9A9D020B.gz>" |
| citation | "Abarenkov, Kessy; Zirk, Allan; Piirmann, Timo; Pöhönen, Raivo; Ivanov, Filipp; Nilsson, R. Henrik; Kõljalg, Urmas (2021): UNITE USEARCH/UTAX release for eukaryotes. UNITE Community. 10.15156/BIO/1280317" |
| fmtscript | "taxref_reformat_sintax.sh" |
| dbversion | "UNITE-alleuk v8.3 (<https://doi.org/10.15156/BIO/1280127>)" |
| unite-alleuk=8.2 |  |
| title | "UNITE USEARCH/UTAX release for eukaryotes - Version 8.2" |
| file |  |
| 0 | "<https://files.plutof.ut.ee/public/orig/7B/B5/7BB51166C988E448392B213A72D4604E2ABB494E20E19E4392F7819FEBFCD036.gz>" |
| citation | "Abarenkov, Kessy; Zirk, Allan; Piirmann, Timo; Pöhönen, Raivo; Ivanov, Filipp; Nilsson, R. Henrik; Kõljalg, Urmas (2020): UNITE USEARCH/UTAX release for eukaryotes. UNITE Community. 10.15156/BIO/786376" |
| fmtscript | "taxref_reformat_sintax.sh" |
| dbversion | "UNITE-alleuk v8.2 (<https://dx.doi.org/10.15156/BIO/786376>)" |
| kraken2_ref_databases |  |
| silva |  |
| title | "Kraken2 pre-formatted SILVA - Version 138" |
| file |  |
| 0 | "<https://genome-idx.s3.amazonaws.com/kraken/16S_Silva138_20200326.tgz>" |
| citation | "<https://www.arb-silva.de/>; Bokulich, N.A., Robeson, M., Dillon, M.R. bokulich-lab/RESCRIPt. Zenodo. <http://doi.org/10.5281/zenodo.3891931>" |
| license | "<https://www.arb-silva.de/silva-license-information/>" |
| fmtscript | "" |
| taxlevels | "D,P,C,O,F,G" |
| silva=138 |  |
| title | "Kraken2 pre-formatted SILVA - Version 138" |
| file |  |
| 0 | "<https://genome-idx.s3.amazonaws.com/kraken/16S_Silva138_20200326.tgz>" |
| citation | "<https://www.arb-silva.de/>; Bokulich, N.A., Robeson, M., Dillon, M.R. bokulich-lab/RESCRIPt. Zenodo. <http://doi.org/10.5281/zenodo.3891931>" |
| license | "<https://www.arb-silva.de/silva-license-information/>" |
| fmtscript | "" |
| taxlevels | "D,P,C,O,F,G" |
| silva=132 |  |
| title | "Kraken2 pre-formatted SILVA - Version 132" |
| file |  |
| 0 | "<https://genome-idx.s3.amazonaws.com/kraken/16S_Silva132_20200326.tgz>" |
| citation | "<https://www.arb-silva.de/>; Bokulich, N.A., Robeson, M., Dillon, M.R. bokulich-lab/RESCRIPt. Zenodo. <http://doi.org/10.5281/zenodo.3891931>" |
| license | "<https://www.arb-silva.de/silva-license-information/>" |
| fmtscript | "" |
| taxlevels | "D,P,C,O,F,G" |
| rdp |  |
| title | "RDP - Ribosomal Database Project - RDP trainset 18/release 11.5" |
| file |  |
| 0 | "<https://genome-idx.s3.amazonaws.com/kraken/16S_RDP11.5_20200326.tgz>" |
| citation | "Cole JR, Wang Q, Fish JA, Chai B, McGarrell DM, Sun Y, Brown CT, Porras-Alfaro A, Kuske CR, Tiedje JM. Ribosomal Database Project: data and tools for high throughput rRNA analysis. Nucleic Acids Res. 2014 Jan;42(Database issue):D633-42. doi: 10.1093/nar/gkt1244. Epub 2013 Nov 27. PMID: 24288368; PMCID: PMC3965039." |
| fmtscript | "" |
| taxlevels | "D,P,C,O,F,G" |
| rdp=18 |  |
| title | "RDP - Ribosomal Database Project - RDP trainset 18/release 11.5" |
| file |  |
| 0 | "<https://genome-idx.s3.amazonaws.com/kraken/16S_RDP11.5_20200326.tgz>" |
| citation | "Cole JR, Wang Q, Fish JA, Chai B, McGarrell DM, Sun Y, Brown CT, Porras-Alfaro A, Kuske CR, Tiedje JM. Ribosomal Database Project: data and tools for high throughput rRNA analysis. Nucleic Acids Res. 2014 Jan;42(Database issue):D633-42. doi: 10.1093/nar/gkt1244. Epub 2013 Nov 27. PMID: 24288368; PMCID: PMC3965039." |
| fmtscript | "" |
| taxlevels | "D,P,C,O,F,G" |
| greengenes |  |
| title | "Kraken2 pre-formatted Greengenes - Version 13.5" |
| file |  |
| 0 | "<https://genome-idx.s3.amazonaws.com/kraken/16S_Greengenes13.5_20200326.tgz>" |
| citation | "McDonald, D., Price, M., Goodrich, J. et al. An improved Greengenes taxonomy with explicit ranks for ecological and evolutionary analyses of bacteria and archaea. ISME J 6, 610–618 (2012). <https://doi.org/10.1038/ismej.2011.139>" |
| fmtscript | "" |
| taxlevels | "D,P,C,O,F,G,S" |
| greengenes=13.5 |  |
| title | "Kraken2 pre-formatted Greengenes - Version 13.5" |
| file |  |
| 0 | "<https://genome-idx.s3.amazonaws.com/kraken/16S_Greengenes13.5_20200326.tgz>" |
| citation | "McDonald, D., Price, M., Goodrich, J. et al. An improved Greengenes taxonomy with explicit ranks for ecological and evolutionary analyses of bacteria and archaea. ISME J 6, 610–618 (2012). <https://doi.org/10.1038/ismej.2011.139>" |
| fmtscript | "" |
| taxlevels | "D,P,C,O,F,G,S" |
| standard |  |
| title | "Standard database - Version 20230605" |
| file |  |
| 0 | "<https://genome-idx.s3.amazonaws.com/kraken/k2_standard_20230605.tar.gz>" |
| citation | "Wood, D. E., Lu, J., & Langmead, B. (2019). Improved metagenomic analysis with Kraken 2. Genome biology, 20(1), 257. <https://doi.org/10.1186/s13059-019-1891-0>" |
| fmtscript | "" |
| taxlevels | "D,P,C,O,F,G,S" |
| standard=20230605 |  |
| title | "Standard database - Version 20230605" |
| file |  |
| 0 | "<https://genome-idx.s3.amazonaws.com/kraken/k2_standard_20230605.tar.gz>" |
| citation | "Wood, D. E., Lu, J., & Langmead, B. (2019). Improved metagenomic analysis with Kraken 2. Genome biology, 20(1), 257. <https://doi.org/10.1186/s13059-019-1891-0>" |
| fmtscript | "" |
| taxlevels | "D,P,C,O,F,G,S" |
| validationSkipDuplicateCheck | false |
| validation-skip-duplicate-check | false |
| validationS3PathCheck | false |
| validation-S3Path-check | false |
| monochromeLogs | false |
| monochrome-logs | false |
