# Supplementary material for: Bacterial microbiome analysis of vaginal, cervical, and endometrial samples in patients with adenomyosis during the window of implantation
Source: Microbiol Spectr. 2026 Feb 18;14(4):e02791-25. doi: 10.1128/spectrum.02791-25 (PMC13055214; doi:10.1128/spectrum.02791-25)
Supplement: Figure S1 — Graphic representation of endometrial sampling. [file spectrum.02791-25-s0002.docx]

**Supplementary Figure S1: Graphic representation of endometrial sampling**


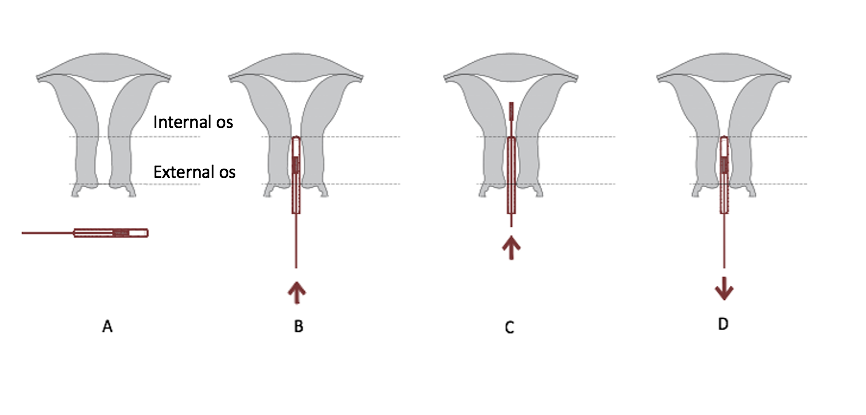


Endometrial sampling description: A – DNA/RNA swab is secured in a sterile sheath. B – The sheath with the endometrial swab is inserted into the cervical canal up to internal cervical os. C – The swab is advanced into the uterine cavity. D – The swab is retracted into the sheath and removed together with the sheath from the cervical canal. The swab is then extracted from the sheath.
