# Supplementary material for: Bacterial microbiome analysis of vaginal, cervical, and endometrial samples in patients with adenomyosis during the window of implantation
Source: Microbiol Spectr. 2026 Feb 18;14(4):e02791-25. doi: 10.1128/spectrum.02791-25 (PMC13055214; doi:10.1128/spectrum.02791-25)
Supplement: Figure S2 — Rarefaction curves. [file spectrum.02791-25-s0003.docx]

**Supplementary Figure S2:** Rarefaction
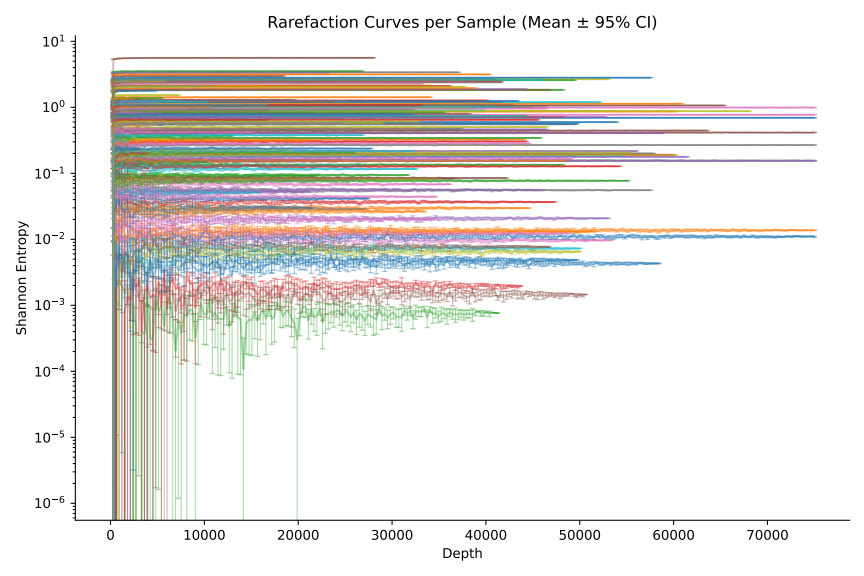
curves show informational saturation of sample diversity, expressed as Shannon entropy, with sequencing depth. Curves are shown for each sequenced sample with errorbars depicting 95% confidence intervals, based on 10-fold resampling at given depths. Shannon diversities were calculated using qiime2 software as part of the nextflow pipeline nf-core/ampliseq v 2.8.0; plots were drawn using the python3 module matplotlib.
